# Supplementary material for: Factors influencing use of essential surgical services in North-East India: a cross-sectional study of obstetric and gynaecological surgery
Source: BMJ Open. 2020 Oct 22;10(10):e038470. doi: 10.1136/bmjopen-2020-038470 (PMC7583072; doi:10.1136/bmjopen-2020-038470)
Supplement: Author's manuscript [file bmjopen-2020-038470.draft_revisions.pdf]

BMJ Open

BMJ Open is committed to open peer review. As part of this commitment we make the peer review history of every article we publish publicly available.

When an article is published we post the peer reviewers' comments and the authors' responses online. We also post the versions of the paper that were used during peer review. These are the versions that the peer review comments apply to.

The versions of the paper that follow are the versions that were submitted during the peer review process. They are not the versions of record or the final published versions. They should not be cited or distributed as the published version of this manuscript.

BMJ Open is an open access journal and the full, final, typeset and author-corrected version of record of the manuscript is available on our site with no access controls, subscription charges or pay-per-view fees (http://bmjopen.bmj.com).

If you have any questions on BMJ Open's open peer review process please email info.bmjopen@bmj.com

BMJ Open

What determines use of surgical services in a low resource setting? Access to obstetric and gynaecological surgery in North-East India

Journal:	*BMJ Open*
Manuscript ID	bmjopen-2020-038470
Article Type:	Original research
Date Submitted by the Author:	12-Mar-2020
Complete List of Authors:	Ensor, Tim; University of Leeds, Nuffield Centre for Health<br>Virk, Amrit; University of Leeds<br>Aruparayil, Noel; University of Leeds
Keywords:	Maternal medicine < OBSTETRICS, Health policy < HEALTH SERVICES ADMINISTRATION & MANAGEMENT, Adult surgery < SURGERY

SCHOLARONE™
Manuscripts

I, the Submitting Author has the right to grant and does grant on behalf of all authors of the Work (as defined in the below author licence), an exclusive licence and/or a non-exclusive licence for contributions from authors who are: i) UK Crown employees; ii) where BMJ has agreed a CC-BY licence shall apply, and/or iii) in accordance with the terms applicable for US Federal Government officers or employees acting as part of their official duties; on a worldwide, perpetual, irrevocable, royalty-free basis to BMJ Publishing Group Ltd ("BMJ") its licensees and where the relevant Journal is co-owned by BMJ to the co-owners of the Journal, to publish the Work in this journal and any other BMJ products and to exploit all rights, as set out in our licence.

The Submitting Author accepts and understands that any supply made under these terms is made by BMJ to the Submitting Author unless you are acting as an employee on behalf of your employer or a postgraduate student of an affiliated institution which is paying any applicable article publishing charge ("APC") for Open Access articles. Where the Submitting Author wishes to make the Work available on an Open Access basis (and intends to pay the relevant APC), the terms of reuse of such Open Access shall be governed by a Creative Commons licence – details of these licences and which Creative Commons licence will apply to this Work are set out in our licence referred to above.

Other than as permitted in any relevant BMJ Author's Self Archiving Policies, I confirm this Work has not been accepted for publication elsewhere, is not being considered for publication elsewhere and does not duplicate material already published. I confirm all authors consent to publication of this Work and authorise the granting of this licence.

For peer review only

Title: What determines use of surgical services in a low resource setting? Access to obstetric and gynaecological surgery in North-East India.

Authors:

Tim Ensor, PhD, University of Leeds, University of Leeds, Worsley Building, Clarendon Way, Leeds, LS2 9NL, + 44 (0) 113 343 6908, t.r.a.ensor@leeds.ac.uk, ORCID ID 0000-0003-0279-9576,

Amrit Virk, PhD, University of Leeds, A.Virk@leeds.ac.uk, ORICD ID 0000-0001-8686-2776

Noel Aruparayil, MBBS, University of Leeds, N.K.Aruparayil@leeds.ac.uk, ORCID ID 0000-0002-2898-772X

Contributorship statement

Tim Ensor conceived and designed the study undertook the data analysis, contributed to interpretation of the results and was wrote the first draft of the paper. **Amrit Virk** helped guide the development of study, interpretation of results and contributed to policy conclusions. She read and edited the final draft. **Noel Aruparauil** provided input on the interpretation of results particularly around clinical aspects of health seeking behaviour. He read and edited the final draft.

-

For peer review only

Abstract: (250 words)

Introduction: There continues to be a large gap between need and actual use of surgery in low-resource settings. While policy frequently focuses on expanding the supply of services, demand-side factors are at least as important in determining under and over use of services. The aim of this study is to understand how these factors influence the use of selected essential obstetric and gynaecological surgical procedures in the underserved and remote setting of North-East India.

Methods: The study combines and makes use of data from a variety of surveys and routine systems. Descriptive analysis of variations in caesarean section, hysterectomy and sterilisation and then multivariate logit analysis of demand and supply-side factors on access to these services is undertaken.

Results: Surgical rates vary substantially both across and within North-East India, correlated with service capacity and socio-economic status. Travel times to surgical facilities are associated with rates of caesarean section and hysterectomy but not sterilisation where services are much more deconcentrated. Travel is less important for surgery in private facilities where capacity is much more dispersed but dominated by the non-poor. The presence of non-doctor medical staff is associated with lower levels of surgical activity.

Conclusion: In low resource, remote settings policy interventions to improve access to service must recognise that surgical rates in low-resource settings are heavily influenced by demand-side factors. As well as boosting services, mechanisms need to mitigate demand-side barriers particularly distance and influence practice to encourage surgical intervention only where clinically indicated.

Funding: National Institute of Health Research

Key words: surgery; obstetrics and gynaecology; maternal health; demand-side barriers; distance

For peer review only

For peer review only - http://bmjopen.bmj.com/site/about/guidelines.xhtml

Article summary

Strengths and limitations of this study

- The study combines three large data sets on obstetric services to allow an exploration of the supply and demand factors affecting use of obstetric and gynaecological services.
- The study demonstrates the important of physical access barriers in contributing to hugely variable surgical rates in an underserved and remote setting
- DHS data does not indicate whether surgery was clinically indicated at an individual level so drawing definitive conclusions on excessive surgery at an individual level is therefore not possible.
- The distance variable is a rough estimate of the actual time to a facility because of the geographic displacement in the GIS variable and the assumption that facilities are based in the centre of the district in the absence of precise GIS coordinates for facilities.

Data sharing statement

No additional data available

Patient and Public Involvement Statement

Patients or the public were not involved in the design, conduct, reporting or dissemination plans of our research.

For peer review only

Introduction

There continues to be a huge difference between user need and use of surgical care in low-resource settings with up to 4.8 billion lacking accessible services.[1,2] Use of services is typified by substantial inequality with both under and over-use of surgical services harming human health[3]. It is estimated that increased appropriate access to surgical services could have a substantial impact on the global burden of disease.[4] Improved surgery is a key component in strengthening a system to deliver universal health coverage.[5,6]

Much recent focus on access to global surgery has been on strengthening the supply side through increasing surgical staff, improving training and infrastructure provision [7,8]. Although a strong supply-side is necessary it is not sufficient for improved access. A host of demand-side factors at the user level such as affordability, proximity to services, socioeconomics and education influence service use.[9] Understanding how these factors influence surgical access is crucial for the design of evidence-based policy interventions yet there is limited evidence on the size of their impact particularly in low resource settings. [2,10,11]

This study aims to understand the extent to which demand and supply factors influence surgical use in such underserved, poorly resourced and remote settings. Our focus is on the eight states in North-East India, one of the most underdeveloped regions in the country with persistently poor health outcomes and substantial access barriers.[12] We examine factors determining use of three essential surgical procedures: caesarean sections, hysterectomy and female sterilisation [13]. Although some of these factors are likely to be specific to this type of surgery others can be regarded as more general. In this respect, these procedures act as tracer services to identify general issues of access to surgical provision.

Methods

Descriptive statistics on state and district level surgical activity in North-East India are compared with other states are presented to set the regional discussion in context. Subsequently, for the eight North-Eastern states (Arunachal Pradesh, Assam, Manipur, Meghalaya, Mizoram, Nagaland, Sikkim and Tripura) multivariate logit analysis is used to examine the association between surgical rates for the three procedures and individual and household characteristics, proximity to and capacity of health services. The economic status of the household is approximated by the asset-based wealth index. Three supply variables are used, available at the village level from the district-level census: doctors, other medical staff and beds expressed as rate per 1,000 population.

For peer review only - http://bmjopen.bmj.com/site/about/guidelines.xhtml

The National Family Health Survey (NFHS) 2015-16 provides a sample of women of reproductive age representative down to district level[14]. The online HMIS provides information on surgical activity and was used to identify centres with substantial surgical capability.[15] The District Census provides supply-side information on numbers of facilities, staff, beds and population by district.[1]

The distance between clusters of households and main surgical centres is computed through a two stage process. The NFHS provides approximate geographical positioning of the cluster from which households are selected. In line with the procedure adopted across DHS surveys, coordinates are then shifted randomly by up to five kilometres in rural and two kilometres in urban areas to protect the anonymity of responding households. HMIS data provides information on the amount of surgical activity in facilities within a district allowing computation of the size of their surgical capacity defined as high (more than 2000 major surgical procedures a year) through to low (less than one per day). The time it takes to travel from the centre of each district to all other districts with high surgical activity is calculated in stata using the georoutei add-in. This takes pairs of coordinates (longitude and latitude) and accesses the HERE mapping database[2] to compute the travel time using available roads adjusted for speed of traffic. We calculate the distance between each district centre and the nearest district with surgical capacity added to the distance between each household cluster and district centre, producing an estimate of total time to a surgical centre.

The data used in this study are all anonymised and in the public domain. DHS was downloaded from the Measure website (dhsprogram.com) while district HMIS and census data are available through public facing websites (censusindia.gov.in; nrhm-mis.nic.in). The funder of the research had no role in study design, collection, analysis and interpretation of data, writing the report or the decision to submit the paper for publication.

Patients or the public were not involved in the design, conduct, reporting or dissemination plans of our research.

Results

Nationally, the HMIS reports a total of 4.3 million (3.1 per 1,000 population) major (with spinal/general anaesthesia) and 11.5 (8.5 per 1,000 population) minor operations in 2017-18. The rates vary from 1.2/1,000 major operations in the country's most populous state Uttar Pradesh to 18/1000 in largely urban city-state Delhi. Rates are much lower than the needs based benchmark of 45/1,000 major operations for South Asia.[1] With the exception of Mizoram state, where health

[1] http://censusindia.gov.in/2011census/dchb/DCHB.html
[2] https://developer.here.com

For peer review only - http://bmjopen.bmj.com/site/about/guidelines.xhtml

service infrastructure appears to be better functioning than in other states,[16] rates of surgery across the North East are at or below the country average of 3.1 per 1,000 population (Figure 1).

For peer review only

For peer review only - http://bmjopen.bmj.com/site/about/guidelines.xhtml

<Figure 1 about here>

The national caesarean section (CS) rate is reported by place of service focused HMIS as 17.5% of live births for 2017-18 while the household focused NFHS reports a similar figure.[14] This average, which is higher than the WHO benchmark for necessary surgical delivery of 10-15% [17], masks substantial state-wide variation ranging from 2.5% in Bihar to over 45% in Tamil Nadu.[15] The NFHS finds 31% of Indian women reporting a sterilisation which is close to the highest rate internationally—33% in the Dominican Republic.[3] Comparing hysterectomy rates across countries is impeded by the nature of the survey data. The NFHS focuses on women of reproductive age where as in established health systems hysterectomy is largely concentrated in older age groups. Studies in India have drawn attention to the relatively high rates of the procedure with one third occurring in women below the age of 40.[18] In NE India rates of gynaecological and obstetric surgery are at or below the national average: CS rates range from 7.9% (Meghalaya) to 21.8% (Tripura) of deliveries (national average=17.5%), hysterectomy from 0.2 (Assam) to 2.8 (Arunachal Pradesh) per 1,000 women (national average=0.8)) and sterilisation 0.2 (Manipur) to 1 (Assam) per 1,000 women (national average=2.6).

<Table 1 about here>

Turning to surgical utilisation, across India use of surgery is positively associated both with the capacity of health services and the wealth of individuals (Table 1) with associations strongest for CS. The stronger association with the total number of registered doctors in the state rather than number of government doctors may reflect the importance of the private sector in delivering the majority of CS across the country. According to the HMIS, 51% of CS were delivered in private facilities in 2016-17 while the NFHS reports 53% for births in 2014-2016. The association with health service capacity and individual wealth is much weaker for hysterectomy and sterilisations. Sterilisation is positively associated with the total number of government doctors but not total registered doctors in states, indicating public sector dominance over the provision of permanent contraception methods with 74% provided in the public sector.[15]

Across NE India, use of surgical services varies widely: CS as a proportion of deliveries by district varies from 0.4% to almost 42% (Interquartile range – IQR - 8.7%), hysterectomy from 0.1% to 4.8% (IQR 1.2%). Rates of female sterilisation vary from 0.5% to more than 21% (IQR 5.7%) (Figure 2).

[3] The rate in Dominican Republic was recorded in the DHS at 33% in 2007, see https://www.statcompiler.com/en/

For peer review only - http://bmjopen.bmj.com/site/about/guidelines.xhtml

<Figure 2 about here>

Surgical use is positively associated with proximity to major surgical centres. CS rates, for example, range from over 25% for clusters than are geographically close to major surgical centres to 5% or lower in areas more than ten hours away (Figure 3). There is a less pronounced relationship between distance and prevalence of hysterectomy (Figure 4) while no clear association is found for sterilisation.

<Figure 3 about here>

<Figure 4 about here>

Turning to the analysis of individual behaviour, all three surgical procedures are positively associated with household wealth (Table 2). This association is strongest for CS and weakest for sterilisation. CS rates are substantially higher for women who are better educated. In contrast, hysterectomy and sterilisation rates are lower for those with secondary education or above compared to those without any education.

<Table 2 about here>

The importance of economic status is likely to be partly the result of cost barriers to obtaining care. Costs of surgical delivery, including transport represent a substantial proportion of per capita income; 11% in the public sector and 28% in the private sector (Table 3). The NFHS found that after distance (36%), treatment cost was the second most important factor (21%) for women not delivering in a facility. In contrast, sterilization costs were reportedly less burdensome for users as 75% of sterilized women in the NFHS reported not paying anything and 48% reported receiving financial compensation.[14]

<Table 3 about here>

The incidence of caesarean section increases with age particularly for those over 30 compared to younger women. The prevalence of hysterectomy and sterilisation is also positively associated with age the latter likely reflecting an increased demand for birth control as family size grows. Higher parity is also associated with greater prevalence of sterilisation. The analysis suggests that women with Muslim or Christian faith are less likely to undergo the three procedures while those living in tribal communities have lower levels of CS than other communities. There is no association with the other gynaecological surgery.

For peer review only

For peer review only - http://bmjopen.bmj.com/site/about/guidelines.xhtml

RSBY (Rashtriya Swasthya Bima Yojana) insurance is the most common insurance in the NE states with the exception of Arunachal Pradesh and Sikkim where state insurance dominates. RSBY membership is negatively associated with caesarean section and has no statistically significant association with hysterectomy. Sterilisation is positively associated with RSBY as well as other types of insurance (Table 2).

Distance to a major surgical centre is negatively associated with both caesarean section and hysterectomy but no statistically significant relationship is found for sterilisation. No information on where sterilisation was carried out is available in the NFHS and those reporting place of hysterectomy is too small for a meaningful analysis. The CS rates vary substantially across the public and private sector for all income groups. Overall CS rates exceed 40% for women delivering in private facilities falling to 11% in public facilities. There is a strongly pronounced income gradient for both facility types (Figure 5).

Proximity to medical services, as measured by doctor numbers, is shown to be positively associated with CS and hysterectomy rates. Availability of more local doctors may also increase referral to facilities even if not situated in the area. Local bed capacity is associated with CS rates but not with hysterectomy. In fact across the eight states, provision of hysterectomy is highly concentrated with just 12% of districts carrying out almost 70% of hysterectomies in 2017/19 (HMIS). Hysterectomy is typically a more complex procedure requiring more resources so it is unsurprising that increased capacity in small local facilities does not influence rates.

<Figure 4 about here>

The association with distance from a main surgical facility is notably different in public and private facilities. For public facilities there is a large and statistically significant difference between women living close versus those living far from a large surgical facility (Figure 5). In contrast, for women delivering in private facilities, the effect of proximity is not nearly so pronounced and not significant for any income group.

Discussion

Surgical rates vary substantially both across and within the states of North-East India, correlated with service capacity and socio-economic status. The results demonstrate the importance of individual level demand-side factors on the use of surgical services across NE India. The strong positive relationship between economic and educational status and CS is evidenced in other studies in South Asia and across LMICs. [19,20]. Like recent studies on hysterectomy in India[18], we find a strong association between hysterectomy rates and household wealth. Higher education, however, is less influential, a finding borne out by another study in India reporting higher hysterectomy rates among

For peer review only - http://bmjopen.bmj.com/site/about/guidelines.xhtml

uneducated, rural women[11]. Turning to sterilisation, economic status is not a strong predictor for prevalence but our results indicate that those with at least secondary education are less likely to be sterilised. Within groups, even after adjusting for distance and socio-economic factors, CS are still higher among non-tribal compared to tribal women, a finding supported by a recent study in western India [21].

With respect to distance as a contributing factor for surgical access, travel times to surgical facilities are associated with CS and hysterectomy rates but not sterilisation. The evident lack of association between distance and sterilisation rates may be explained by the greater availability of this procedure at smaller facilities and surgical outreach camps; the 2015-16 DHS reports that in rural areas 53% of sterilisation is provided at a level below a government municipal or main private hospital.[14] Surgery in private facilities where capacity is much more dispersed and dominated by non-poor users is less contingent on travel time. This may be linked to the greater number of smaller private facilities that offer surgery so that travel-time is less important. Rates of CS are substantially above the 10-15% level even for the poorest income groups. These higher rates may result partly from women with more complex deliveries seeking care from these facilities but also an excess preference for CS over non-surgical delivery in private facilities, a concern raised in other studies across India [22].

We find the presence of non-doctor medical staff is generally associated with lower levels of surgical activity. This is supported by other findings: across widely different contexts - for example the US and Iran [23,24] - there is evidence that surgical delivery is lower where more care is provided by nurses and midwives. Our study supports this with lower rates of both CS and sterilisation where similar services can be provided by non-surgical alternatives.

The negative association between membership of the RSBY scheme and CS and lack of association with hysterectomy and CS surgery and the state insurance scheme, also found in other studies, [25] remains puzzling given that both form part of the respective insurance packages. One explanation is that since RSBY is largely a scheme for the poor, the negative effect of lower income over-ride any positive impact of holding insurance. Another explanation is that beneficiaries are unware of benefits of insurance. This was suggested in a recent study of RSBY which revealed that lack of awareness of scheme benefits resulted in RSBY beneficiaries accessing paid, private services rather than through RSBY facilities [26]. A further explanation may be that the reimbursement rates for surgery across the states are lower than the costs of services making facilities less likely to accept insured patients. Delays and other issues with the insurance mechanisms also often make private providers reluctant to participate in schemes. [26] The findings suggest that access to surgery is not

For peer review only - http://bmjopen.bmj.com/site/about/guidelines.xhtml

guaranteed by inclusion in an insurance benefits package. It is also important to ensure that full costs of delivering services are properly compensated particularly for providers that are delivering services to more vulnerable groups.

The study has a number of limitations. The self-reported DHS data does not indicate whether surgery was clinically indicated at an individual level so drawing definitive conclusions on excessive surgery at an individual level is therefore not possible. Nevertheless, the population estimates suggest rates that for some groups are too high and some too low. The distance variable is a rough estimate of the actual time to a facility because of the geographic displacement in the GIS variable and the assumption that facilities are based in the centre of the district in the absence of precise GIS coordinates for facilities. Finally, the study takes no account of the quality of service provided in facilities, a variable that in itself is likely to be an important but missing determinant of use of services.

The huge variation in access to gynaecological and obstetric surgery across North-East India illustrates the persistent importance of factors that both discourage essential surgical care and incentivise some unnecessary surgery. Evidence from a range of contexts suggest that supply-side improvements will not increase use of services without mitigating demand-barriers.[27] Cost-effective solutions to alleviate these barriers might either take the form of increasing services in remoter areas (e.g. through outreach services plus rural case finding as practised in other contexts[28]), increasing the ability of vulnerable groups to access geographically more accessible private services at low cost, or attempting to compensate for the greater travel costs of those living in remoter areas. Elsewhere in South Asia, such compensation has been shown to be important in increasing service use.[29,30] Consideration might be given to incorporating such compensation into financing mechanisms.

While increasing access to services it is important for some, for others policy needs to focus on limiting excessive surgery. For CS and hysterectomy high rates are present for richer and more educated groups but for sterilisation higher use is evident for those with less education. Policy to reduce unnecessary surgery in rich and poor households requires ensuring adequate and accessible staff to promote and undertake non-surgical alternatives in both rural and urban areas. At the same time, further attention is required to ensure that surgery is accessible when required by mitigating demand-side barriers for those with clear clinical need.

Acknowledgement: This research was funded by the National Institute for Health Research (NIHR) (16/137/44) using UK aid from the UK Government to support global health research. The views

For peer review only - http://bmjopen.bmj.com/site/about/guidelines.xhtml

expressed in this publication are those of the author(s) and not necessarily those of the NIHR or the UK Department of Health and Social Care.

For peer review only

For peer review only - http://bmjopen.bmj.com/site/about/guidelines.xhtml

References

1. Rose J, Weiser TG, Hider P, Wilson L, Gruen RL, Bickler SW. Estimated need for surgery worldwide based on prevalence of diseases: a modelling strategy for the WHO Global Health Estimate. *The Lancet Global health* 2015; **3 Suppl 2**: S13-20.

2. Alkire BC, Raykar NP, Shrime MG, et al. Global access to surgical care: a modelling study. *The Lancet Global health* 2015; **3**(6): e316-23.

3. Brownlee S, Chalkidou K, Doust J, et al. Evidence for overuse of medical services around the world. *Lancet* 2017; **390**(10090): 156-68.

4. Bae JY, Groen RS, Kushner AL. Surgery as a public health intervention: common misconceptions versus the truth. *Bull World Health Organ* 2011; **89**(6): 394.

5. Okoroh JS, Chia V, Oliver EA, Dharmawardene M, Riviello R. Strengthening Health Systems of Developing Countries: Inclusion of Surgery in Universal Health Coverage. *World J Surg* 2015; **39**(8): 1867-74.

6. Mock CN, Donkor P, Gawande A, et al. Essential surgery: key messages from Disease Control Priorities, 3rd edition. *Lancet* 2015; **385**(9983): 2209-19.

7. Meara JG, Leather AJ, Hagander L, et al. Global Surgery 2030: Evidence and solutions for achieving health, welfare, and economic development. *Surgery* 2015; **158**(1): 3-6.

8. Raykar NP, Yorlets RR, Liu C, et al. A qualitative study exploring contextual challenges to surgical care provision in 21 LMICs. *Lancet* 2015; **385 Suppl 2**: S15.

9. Ensor T, Cooper S. Overcoming barriers to health service access: influencing the demand side. *Health Policy Planning* 2004; **19**(2): 69-79.

10. Grimes CE, Bowman KG, Dodgion CM, Lavy CB. Systematic review of barriers to surgical care in low-income and middle-income countries. *World J Surg* 2011; **35**(5): 941-50.

11. Shekhar C, Paswan B, Singh A. Prevalence, sociodemographic determinants and self-reported reasons for hysterectomy in India. *Reprod Health* 2019; **16**(1): 118.

12. Press Information Bureau. North Eastern Region Vision 2020 (Press Release) Delhi: Government of India; 2019.

13. Debas HT, Donkor P, Gawande A, Jamison D, Kruk M, Mock C. Essential Surgery. Third ed. Washington: World Bank Group; 2015.

14. International Institute for Population Sciences. National Health and Family Survey 2015-16. India, Mubai: IIPS, 2017.

15. Government of India. Health Management Information System. 2019. https://nrhm-mis.nic.in/SitePages/Home.aspx.

16. Saikia D, Kangkana Das K. Rural Health Infratsutures in the North-East India. Munich: Munich Personal RePEc Archive, 2012.

17. Guilmoto CZ, Dumont A. Trends, Regional Variations, and Socioeconomic Disparities in Cesarean Births in India, 2010-2016. *JAMA Netw Open* 2019; **2**(3): e190526.

18. Prusty RK, Choithani C, Gupta SD. Predictors of hysterectomy among married women 15-49 years in India. *Reprod Health* 2018; **15**(1): 3.

19. Neuman M, Alcock G, Azad K, et al. Prevalence and determinants of caesarean section in private and public health facilities in underserved South Asian communities: cross-sectional analysis of data from Bangladesh, India and Nepal. *BMJ open* 2014; **4**(12): e005982.

20. Boatin AA, Schlotheuber A, Betran AP, et al. Within country inequalities in caesarean section rates: observational study of 72 low and middle income countries. *BMJ* 2018; **360**: k55.

21. Desai G, Anand A, Modi D, et al. Rates, indications, and outcomes of caesarean section deliveries: A comparison of tribal and non-tribal women in Gujarat, India. *PLoS One* 2017; **12**(12): e0189260.

22. Singh P, Hashmi G, Swain PK. High prevalence of cesarean section births in private sector health facilities- analysis of district level household survey-4 (DLHS-4) of India. *BMC Public Health* 2018; **18**(1): 613.

For peer review only - http://bmjopen.bmj.com/site/about/guidelines.xhtml

23. Kashanian M, Javadi F, Haghighi MM. Effect of continuous support during labor on duration of labor and rate of cesarean delivery. *Int J Gynaecol Obstet* 2010; **109**(3): 198-200.
24. Lydon-Rochelle M. Cesarean delivery rates in women cared for by certified nurse-midwives in the United States: a review. *Birth* 1995; **22**(4): 211-9.
25. Desai S, Shuka A, Nambiar D, Ved R. Patterns of hysterectomy in India: a national and state-level analysis of the Fourth National Family Health Survey (2015-2016). *BJOG* 2019; **126 Suppl 4**: 72-80.
26. Yadav V, Kumar S, Balasubramaniam S, et al. Facilitators and barriers to participation of private sector health facilities in government-led schemes for maternity services in India: a qualitative study. *BMJ open* 2017; **7**(6): e017092.
27. O'Donnell O. Access to health care in developing countries: breaking down demand side barriers. *Cad Saude Publica* 2007; **23**: 2820 - 34.
28. White MC, Hamer M, Biddell J, et al. Facilitating access to surgical care through a decentralised case-finding strategy: experience in Madagascar. *BMJ Glob Health* 2017; **2**(3): e000427.
29. Ensor T, Bhatt H, Tiwari S. Incentivizing universal safe delivery in Nepal: 10 years of experience. *Health Policy Plan* 2017; **32**(8): 1185-92.
30. De Costa A, Patil R, Kushwah SS, Diwan VK. Financial incentives to influence maternal mortality in a low-income setting: making available 'money to transport' - experiences from Amarpatan, India. *Glob Health Action* 2009; **2**.

For peer review only

For peer review only - http://bmjopen.bmj.com/site/about/guidelines.xhtml

Tables

Table 1: Correlation between surgery, income per capita and supply side variables across all states of India

	Operations per 1,000 population	C-section rate (% live births)	Hysterectomy per 1,000 women	Sterilisations per 1,000 women
All India	3.1	17%	0.8	2.5
NE India States	2.8	18%	0.5	0.8
**R-squared statistics**				
Registered doctors per 1,000	0.373***	0.612***	0.179**	0.003
Government doctors per 1,000	0.281**	0.234**	0.102*	0.252**
Beds per 1,000	0.288**	0.336**	0.090*	0.094*
GDP per capita	0.103*	0.280**	0.047	0.020

Note: significant at the *** 1% level, ** 5% level, * 10% level.

For peer review only

For peer review only - http://bmjopen.bmj.com/site/about/guidelines.xhtml

Table 2: Association between use of surgical services and individual, household and supply side characteristics (Multivariate logit with cluster adjusted standard errors)

	Caesarean Section			Hysterectomy			Sterilisation		
	Coef.	t-statistic	sig.	Coef.	t-statistic	sig.	Coef.	t-statistic	sig.
**Wealth group (base=poorest)**									
poorer	0.483	3.840	***	0.495	2.480	**	0.013	0.190	
middle	1.093	8.390	***	0.739	3.610	***	0.105	1.380	
richer	1.629	11.660	***	1.162	5.480	***	0.184	2.010	**
richest	2.072	13.340	***	1.330	5.480	***	0.459	3.740	***
	-	-		-	-		-	-	
**Education (base=no education)**	-	-		-	-		-	-	
Completed primary	0.552	3.660	***	0.301	1.900	*	- 0.012	- 0.170	
Completed secondary	0.834	6.370	***	- 0.080	- 0.590		- 0.382	- 5.460	***
Completed Higher	1.230	7.950	***	- 0.253	- 0.980		- 0.931	- 7.100	***
	-	-		-	-		-	-	
**Parity (base=1 delivery)**	-	-		-	-		-	-	
	-	-		-	-		-	-	
Two	- 0.641	- 9.060	***	0.136	0.780		2.432	18.630	***
Three	- 1.291	- 12.190	***	0.162	0.880		2.914	21.090	***
Four or more	- 1.882	- 13.170	***	- 0.145	- 0.780		2.823	19.450	***
	-	-		-	-		-	-	
**Marital status (base=never married)**	-	-		-	-		-	-	
married	- 0.763	- 1.380		1.074	2.400	**	1.078	1.350	
widowed	- 0.875	- 1.360		0.579	1.180		0.424	0.530	
divorced	- 0.694	- 1.050		1.233	2.140	**	0.473	0.560	
living together/separated	- 0.999	- 1.530		0.108	0.170		0.619	0.730	
	-	-		-	-		-	-	
**Religion (base=hindu)**	-	-		-	-		-	-	
muslim	- 0.660	- 5.840	***	- 0.723	- 3.230	***	- 1.649	- 14.340	***
christian	- 0.281	- 2.510	**	- 0.014	- 0.070		- 0.298	- 2.850	***
sikh	3.705	3.090	***	-	-		0.273	0.200	
buddhist/neo-buddhist	- 0.671	- 3.850	***	0.347	0.850		- 0.225	- 1.350	
jain	- 1.851	- 1.320		3.264	2.670	***	1.336	2.190	**
no religion	0.436	0.740		- 0.086	- 0.110		- 1.007	- 2.540	**
other	- 0.111	- 0.860		0.230	1.020		- 1.077	- 9.420	***
	-	-		-	-		-	-	

For peer review only

For peer review only - http://bmjopen.bmj.com/site/about/guidelines.xhtml

	Caesarean Section			Hysterectomy			Sterilisation		
	Coef.	t-statistic	sig.	Coef.	t-statistic	sig.	Coef.	t-statistic	sig.
**Caste/Tribe (base=none)**	-	-		-	-		-	-	
Caste	- 0.151	- 1.360		- 0.100	- 0.530		0.165	1.620	
Tribe	- 0.562	- 4.100	***	- 0.259	- 0.970		- 0.148	- 1.150	
	-	-		-	-		-	-	
**Age group (base=15-19)**	-	-		-	-		-	-	
20-25	0.589	3.460	***	- 2.426	- 3.740	***	0.869	1.190	
25-30	0.913	5.260	***	- 1.229	- 4.290	***	1.289	1.800	*
30-35	1.311	7.190	***	0.103	0.510		1.671	2.320	**
35-40	1.589	8.340	***	0.821	5.430	***	1.810	2.500	**
40-45	1.613	6.630	***	0.529	4.360	***	1.715	2.380	**
45-50	1.371	2.550	**	-	-		1.607	2.220	**
								-	
**Type of insurance (base=no insurance)**								-	
state	0.072	0.580		0.017	0.120		0.226	2.550	**
rsby	- 0.254	- 2.170	**	0.092	0.470		0.266	3.550	***
Other insurance	0.252	1.100		0.008	0.020		0.434	2.800	***
	-	-		-	-		-	-	
**Supply-side variables**	-	-		-	-		-	-	
Drs/1,000 population	0.022	1.670	*	- 0.043	- 1.670	*	0.031	3.090	***
Other medical/1,000 population	- 0.021	- 2.930	***	0.011	0.990		- 0.044	- 7.050	***
Time to main surgical facility	- 0.001	- 3.550	***	- 0.001	- 2.340	**	- 0.000	- 0.710	
	-	-		-	-		-	-	
Beds/1,000 population	0.017	3.690	***	0.005	0.720		0.025	6.620	***
	-	-		-	-		-	-	
Constant	- 2.494	- 4.120	***	-	-		- 6.695	- 6.320	***

Note: Significant at the *** 1% level, ** 5% level, * 10% level.

For peer review only

For peer review only - http://bmjopen.bmj.com/site/about/guidelines.xhtml

Table 3: costs of delivery care (US dollars and % of per capita income)

	Public	Private	Total	Public	Private	Total
**Non-surgical delivery**	Cost per delivery			% of per capita income		
Facility	$ 38	$ 140	$ 50	3.3%	12.0%	4.3%
Transport	$ 5	$ 9	$ 6	0.4%	0.8%	0.5%
Total	$ 44	$ 148	$ 56	3.8%	12.8%	4.8%
**Surgical delivery**						
Facility	$ 120	$ 311	$ 199	10.3%	26.8%	17.1%
Transport	$ 8	$ 15	$ 11	0.7%	1.3%	0.9%
Total	$ 128	$ 325	$ 210	11.0%	28.0%	18.1%

Note: state-wise GDP obtained from http://mospi.nic.in/data

For peer review only

For peer review only - http://bmjopen.bmj.com/site/about/guidelines.xhtml

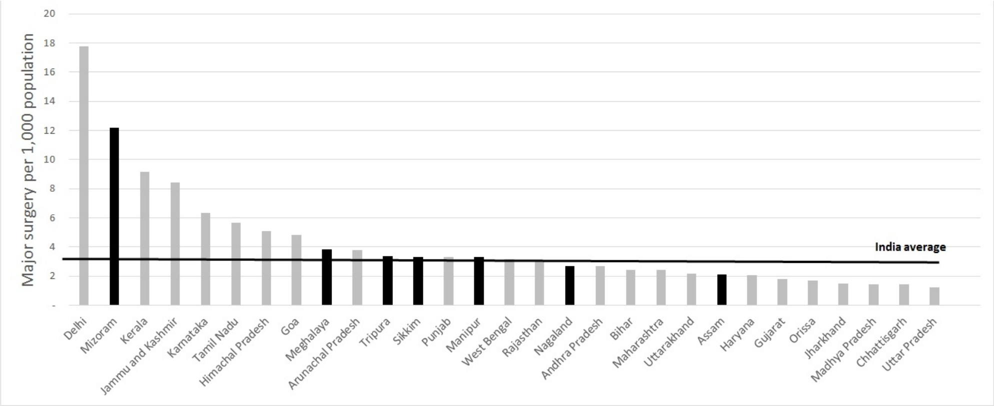

278x113mm (120 x 120 DPI)

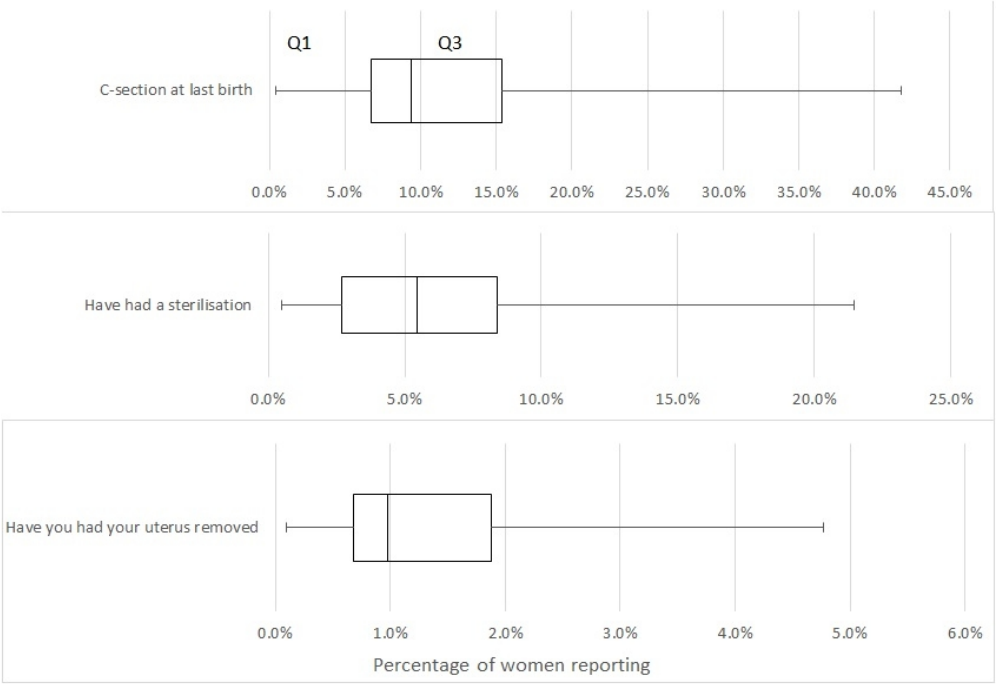

179x123mm (120 x 120 DPI)

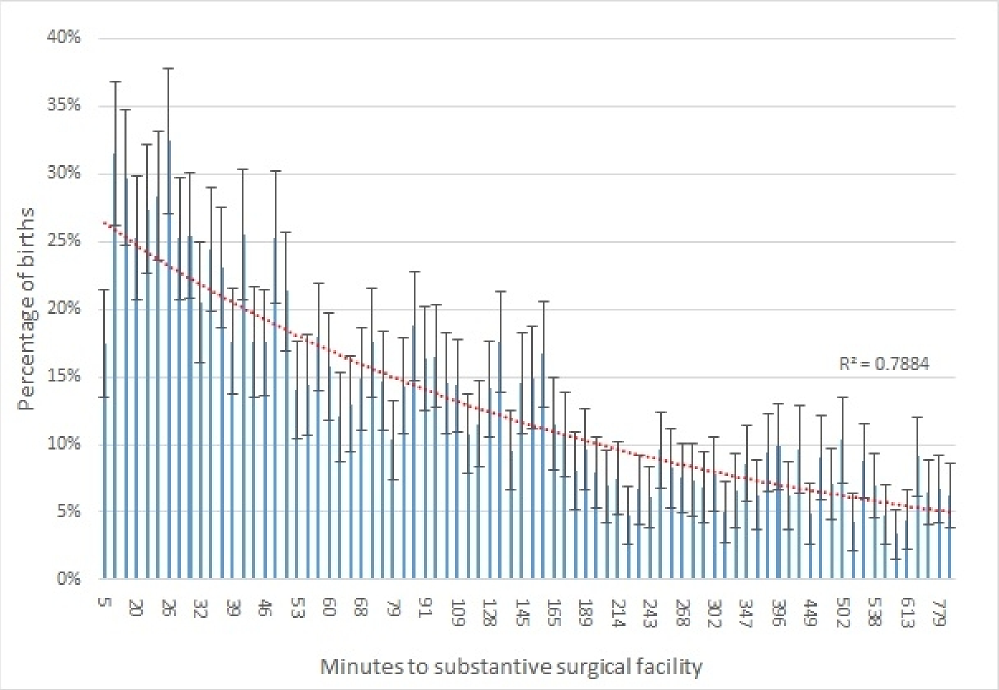

128x88mm (120 x 120 DPI)

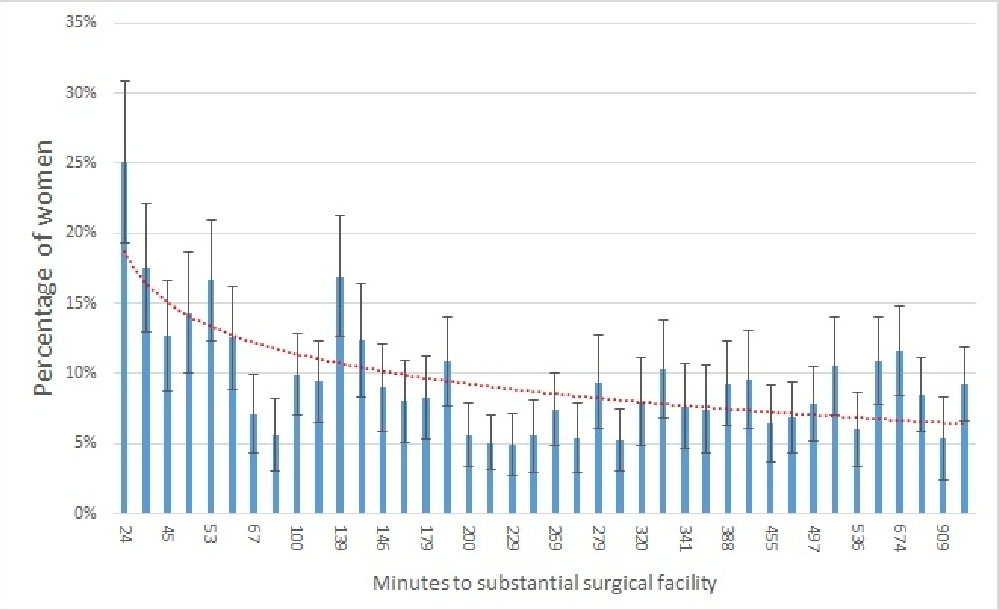

139x85mm (120 x 120 DPI)

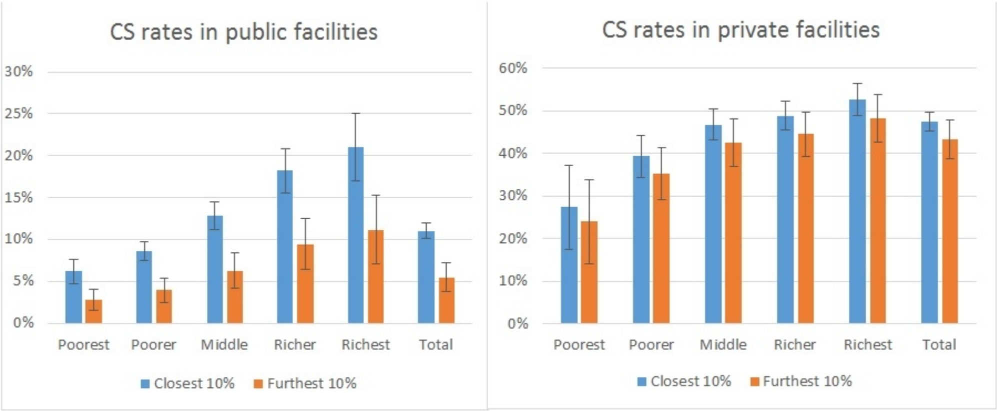

190x79mm (120 x 120 DPI)

BMJ Open

What determines use of surgical services in a low resource setting? Access to obstetric and gynaecological surgery in North-East India

Journal:	*BMJ Open*
Manuscript ID	bmjopen-2020-038470.R1
Article Type:	Original research
Date Submitted by the Author:	29-Jul-2020
Complete List of Authors:	Ensor, Tim; University of Leeds, Nuffield Centre for Health<br>Virk, Amrit; University of Leeds<br>Aruparayil, Noel; University of Leeds
**Primary Subject Heading**:	Global health
Secondary Subject Heading:	Health policy, Public health
Keywords:	Maternal medicine < OBSTETRICS, Health policy < HEALTH SERVICES ADMINISTRATION & MANAGEMENT, Adult surgery < SURGERY

SCHOLARONE™
Manuscripts

I, the Submitting Author has the right to grant and does grant on behalf of all authors of the Work (as defined in the below author licence), an exclusive licence and/or a non-exclusive licence for contributions from authors who are: i) UK Crown employees; ii) where BMJ has agreed a CC-BY licence shall apply, and/or iii) in accordance with the terms applicable for US Federal Government officers or employees acting as part of their official duties; on a worldwide, perpetual, irrevocable, royalty-free basis to BMJ Publishing Group Ltd ("BMJ") its licensees and where the relevant Journal is co-owned by BMJ to the co-owners of the Journal, to publish the Work in this journal and any other BMJ products and to exploit all rights, as set out in our licence.

The Submitting Author accepts and understands that any supply made under these terms is made by BMJ to the Submitting Author unless you are acting as an employee on behalf of your employer or a postgraduate student of an affiliated institution which is paying any applicable article publishing charge ("APC") for Open Access articles. Where the Submitting Author wishes to make the Work available on an Open Access basis (and intends to pay the relevant APC), the terms of reuse of such Open Access shall be governed by a Creative Commons licence – details of these licences and which Creative Commons licence will apply to this Work are set out in our licence referred to above.

Other than as permitted in any relevant BMJ Author's Self Archiving Policies, I confirm this Work has not been accepted for publication elsewhere, is not being considered for publication elsewhere and does not duplicate material already published. I confirm all authors consent to publication of this Work and authorise the granting of this licence.

For peer review only

Title: What determines use of surgical services in a low resource setting? Access to obstetric and gynaecological surgery in North-East India.

Authors:

Tim Ensor, PhD, University of Leeds, University of Leeds, Worsley Building, Clarendon Way, Leeds, LS2 9NL, + 44 (0) 113 343 6908, t.r.a.ensor@leeds.ac.uk, ORCID ID 0000-0003-0279-9576,

Amrit Virk, PhD, University of Leeds, A.Virk@leeds.ac.uk, ORICD ID 0000-0001-8686-2776

Noel Aruparayil, MBBS, University of Leeds, N.K.Aruparayil@leeds.ac.uk, ORCID ID 0000-0002-2898-772X

For peer review only

-

For peer review only - http://bmjopen.bmj.com/site/about/guidelines.xhtml

Abstract: (250 words)

Introduction: There continues to be a large gap between need and actual use of surgery in low-resource settings. While policy frequently focuses on expanding the supply of services, demand-side factors are at least as important in determining under and over utilisation. The aim of this study is to understand how these factors influence the use of selected essential obstetric and gynaecological surgical procedures in the underserved and remote setting of North-East India.

Methods: The study combines and makes use of data from a variety of surveys and routine systems. Descriptive analysis of variations in caesarean section, hysterectomy and sterilisation and then multivariate logit analysis of demand and supply-side factors on access to these services is undertaken.

Results: Surgical rates vary substantially both across and within North-East India, correlated with service capacity and socio-economic status. Travel times to surgical facilities are associated with rates of caesarean section and hysterectomy but not sterilisation where services are much more deconcentrated. Travel is less important for surgery in private facilities where capacity is much more dispersed but dominated by the non-poor. The presence of non-doctor medical staff is associated with lower levels of surgical activity.

Conclusion: In low resource, remote settings policy interventions to improve access to services must recognise that surgical rates in low-resource settings are heavily influenced by demand-side factors. As well as boosting services, mechanisms need to mitigate demand-side barriers particularly distance and influence practice to encourage surgical intervention only where clinically indicated.

Funding: National Institute of Health Research

Key words: surgery; obstetrics and gynaecology; maternal health; demand-side barriers; distance

For peer review only

For peer review only - http://bmjopen.bmj.com/site/about/guidelines.xhtml

Article summary

Strengths and limitations of this study

- Combines three large data sets on obstetric services to allow an exploration of the supply and demand factors affecting use of obstetric and gynaecological services.
- Makes use of a distance variable that allows consideration of the effect of location on demand for services
- The distance variable is a rough estimate of the actual time to a facility because of the geographic displacement in the GIS variable and the assumption that facilities are based in the centre of the district in the absence of precise GIS coordinates for facilities.
-
- DHS data does not indicate whether surgery was clinically indicated at an individual level so drawing definitive conclusions on excessive surgery at an individual level is therefore not possible.
- Relies on self-reported use of surgical services.

Patient and Public Involvement Statement

Patients or the public were not involved in the design, conduct, reporting or dissemination plans of our research.

For peer review only

For peer review only - http://bmjopen.bmj.com/site/about/guidelines.xhtml

Introduction

There continues to be a huge difference between user need and use of surgical care in low-resource settings with up to 4.8 billion lacking accessible services.[1,2] Use of services is typified by substantial inequality with both under and over-use of surgical services harming human health[3]. It is estimated that increased appropriate access to surgical services could have a substantial impact on the global burden of disease.[4] Improved surgery is a key component in strengthening a system to deliver universal health coverage.[5,6]

Much recent focus on access to global surgery has been on strengthening the supply side through increasing surgical staff, improving training and infrastructure provision [7,8]. A strong supply-side is necessary but it is not sufficient for improved access. A host of demand-side factors at the user level such as affordability, proximity to services, socioeconomics and education influence service use.[9] Understanding how these factors influence surgical access is crucial for the design of evidence-based policy interventions yet there is limited evidence on the size of their impact particularly in low resource settings. [2,10,11]

This study aims to examine the extent to which demand and supply factors influence surgical use in such underserved, poorly resourced and remote settings. Our focus is on the eight states in North-East India, one of the most underdeveloped regions in the country with persistently poor health outcomes and substantial access barriers.[12] We examine factors determining use of three essential surgical procedures: caesarean sections, hysterectomy and female sterilisation [13]. Although some of these factors are likely to be specific to this type of surgery others can be regarded as more general. In this respect, these procedures act as tracer services to identify general issues of access to surgical provision.

Methods

Data sources: The study makes use of three data sources. The National Family Health Survey (NFHS) 2015-16 provides a sample of women of reproductive age that is statistically representative down to district level[14]. The online HMIS provides information on surgical activity and was used to identify centres with surgical capability.[15] The District Census provides supply-side information on numbers of facilities, staff, beds and population by district.[16]

Variables and method of analysis: Descriptive statistics on state and district level surgical activity in North-East India are compared with other states and presented to set the regional discussion in context. Subsequently, for the eight North-Eastern states (Arunachal Pradesh, Assam, Manipur, Meghalaya, Mizoram, Nagaland, Sikkim and Tripura) multivariate logit analysis is used to examine

For peer review only - http://bmjopen.bmj.com/site/about/guidelines.xhtml

the association between surgical rates for the three procedures, individual and household characteristics (age, education, parity, caste/tribe, marital status, insurance held and economic status), proximity to and capacity of health services. The economic status of the household is approximated by the asset-based wealth index.[17] Three supply variables are used, available at the village level from the district-level census: doctors, other medical staff and beds expressed as rate per 1,000 population.

The distance between clusters of households and main surgical centres is computed through a two stage process. The NFHS provides approximate geographical positioning of the cluster from which households are selected. In line with the procedure adopted across DHS surveys, coordinates are then shifted randomly by up to five kilometres in rural and two kilometres in urban areas to protect the anonymity of responding households. HMIS data provides information on the amount of surgical activity in facilities within a district allowing computation of the size of their surgical capacity defined as high (more than 2000 major surgical procedures a year) through to low (less than one per day). The time it takes to travel from the centre of each district to all other districts with high surgical activity is calculated in Stata using the georoutei add-in.[18] This takes pairs of coordinates (longitude and latitude) and accesses the HERE mapping database (developer.here.com) to compute the total travel time using available roads adjusted for speed of traffic. We calculated the distance between each district centre and the nearest district with surgical capacity which was then added to the distance between each household cluster and district centre, producing an estimate of total time to a surgical centre.

Ethical considerations: The data used in this study are all anonymised and in the public domain and no additional ethics approval was required in order to undertake the study. DHS was downloaded from the Measure website (dhsprogram.com) while district HMIS and census data are available through public facing websites (censusindia.gov.in; nrhm-mis.nic.in). The funder of the research had no role in the design of the surveys used (NFHS, HMIS or District Census) nor any role in the , analysis and interpretation of data, writing the report or the decision to submit the paper for publication. Patients or the public were not involved in the design, conduct, reporting or dissemination plans of our research

Results

Nationally, the HMIS reports a total of 4.3 million (3.1 per 1,000 population) major (with spinal/general anaesthesia) and 11.5 million (8.5 per 1,000 population) minor surgical procedures in 2017-18. The rates vary from 1.2/1,000 major surgical procedures in the country's most populous state Uttar Pradesh to 18/1000 in largely urban city-state Delhi. Rates are much lower than the

For peer review only - http://bmjopen.bmj.com/site/about/guidelines.xhtml

needs based benchmark of 45/1,000 major surgical procedures for South Asia.[1] With the exception of Mizoram state, where health service infrastructure appears to be better functioning than in other states,[19] rates of surgery across the North East are at or below the country average of 3.1 per 1,000 population (Figure 1).

For peer review only

For peer review only - http://bmjopen.bmj.com/site/about/guidelines.xhtml

<Figure 1 about here>

The national caesarean section (CS) rate is reported by place of service focused HMIS as 17.5% of live births for 2017-18 while the household focused NFHS reports a similar figure.[14] This average, which is higher than the WHO benchmark for necessary surgical delivery of 10-15% [20], masks substantial state-wide variation ranging from 2.5% in Bihar to over 45% in Tamil Nadu.[15] The NFHS reported 31% of Indian women that had a sterilisation which is close to the highest rate internationally—33% in the Dominican Republic.[21] Comparing hysterectomy rates across countries is impeded by the nature of the survey data. The NFHS focuses on women of reproductive age where as in established health systems hysterectomy is largely concentrated in older age groups. Studies in India have drawn attention to the relatively high rates of the procedure with one third occurring in women below the age of 40.[22] In NE India rates of gynaecological and obstetric surgery are at or below the national average: CS rates range from 7.9% (Meghalaya) to 21.8% (Tripura) of deliveries (national average=17.5%), hysterectomy from 0.2 (Assam) to 2.8 (Arunachal Pradesh) per 1,000 women (national average=0.8)) and sterilisation 0.2 (Manipur) to 1 (Assam) per 1,000 women (national average=2.6).

<Table 1 about here>

Turning to surgical utilisation, across India use of surgery (per 1,000 population) is positively associated both with the capacity of health services and the wealth of individuals (Table 1) with associations strongest for CS. The stronger association with the total number of registered doctors (per 1,000 population) in the state rather than number of government doctors (per 1,000 population) may reflect the importance of the private sector in delivering the majority of CS across the country. According to the HMIS, 51% of CS were delivered in private facilities in 2016-17 while the NFHS reports 53% for births in 2014-2016. The association with health service capacity and individual wealth is much weaker for hysterectomy and sterilisations. Sterilisation (per 1,000 women) is positively associated with the total number of government doctors but not total registered doctors in states, indicating public sector dominance over the provision of permanent contraception methods with 74% provided in the public sector.[15]

Across NE India, use of surgical services varies widely: CS as a proportion of deliveries by district varies from 0.4% to almost 42% (Interquartile range – IQR - 8.7%), hysterectomy from 0.1% to 4.8% (IQR 1.2%). Rates of female sterilisation vary from 0.5% to more than 21% (IQR 5.7%) (Figure 2).

For peer review only - http://bmjopen.bmj.com/site/about/guidelines.xhtml

<Figure 2 about here>

Surgical use is positively associated with proximity to major surgical centres. CS rates, for example, range from over 25% for clusters than are geographically close to major surgical centres to 5% or lower in areas more than ten hours away (Figure 3). There is a less pronounced relationship between distance and prevalence of hysterectomy (Figure 4) while no clear association is found for sterilisation.

<Figure 3 about here>

<Figure 4 about here>

Turning to the analysis of individual behaviour, all three surgical procedures are positively associated with household wealth (Table 2). This association is strongest for CS and weakest for sterilisation. CS rates are substantially higher for women who are better educated. In contrast, hysterectomy and sterilisation rates are lower for those with secondary education or above compared to those without any education.

<Table 2 about here>

The importance of economic status is likely to be partly the result of cost barriers to obtaining care. Costs of surgical delivery, including transport represent a substantial proportion of per capita income; 11% in the public sector and 28% in the private sector (Table 3). The NFHS found that after distance (36%), treatment cost was the second most important factor (21%) for women not delivering in a facility. In contrast, sterilization costs were reportedly less burdensome for users as 75% of sterilized women in the NFHS reported not paying anything and 48% reported receiving financial compensation.[14]

<Table 3 about here>

The incidence of caesarean section increases with age particularly for those over 30 compared to younger women. The prevalence of hysterectomy and sterilisation is also positively associated with age the latter likely reflecting an increased demand for birth control as family size grows. Higher parity is also associated with greater prevalence of sterilisation. The analysis suggests that women with Muslim or Christian faith are less likely to undergo the three procedures while those living in tribal communities have lower levels of CS than other communities. There is no association with the other gynaecological surgery.

For peer review only - http://bmjopen.bmj.com/site/about/guidelines.xhtml

For peer review only

RSBY (Rashtriya Swasthya Bima Yojana) insurance is the most common insurance in the NE states with the exception of Arunachal Pradesh and Sikkim where state insurance dominates. RSBY membership is negatively associated with caesarean section and has no statistically significant association with hysterectomy. Sterilisation is positively associated with RSBY as well as other types of insurance (Table 2).

Distance to a major surgical centre is negatively associated with both caesarean section and hysterectomy but no statistically significant relationship is found for sterilisation. No information on where sterilisation was carried out is available in the NFHS and those reporting place of hysterectomy is too small for a meaningful analysis. The CS rates vary substantially across the public and private sector for all income groups. Overall CS rates exceed 40% for women delivering in private facilities falling to 11% in public facilities. There is a strongly pronounced income gradient for both facility types (Figure 5).

Proximity to medical services, as measured by doctor numbers, is shown to be positively associated with CS and hysterectomy rates. Availability of more local doctors may also increase referral to facilities even if not situated in the area. Local bed capacity is associated with CS rates but not with hysterectomy. In fact across the eight states, provision of hysterectomy is highly concentrated with just 12% of districts carrying out almost 70% of hysterectomies in 2017/19 (HMIS).

<Figure 4 about here>

The association with distance from a main surgical facility is notably different in public and private facilities. For public facilities there is a large and statistically significant difference between women living close versus those living far from a large surgical facility (Figure 5). In contrast, for women delivering in private facilities, the effect of proximity is not nearly so pronounced and not significant for any income group.

Discussion

Key findings: Surgical rates vary substantially both across and within the states of North-East India, correlated with service capacity and socio-economic status. The results demonstrate the importance of individual level demand-side factors on the use of surgical services across NE India. The strong positive relationship between economic and educational status and CS is evidenced in other studies in South Asia and across LMICs. [23,24]. Like recent studies on hysterectomy in India[22], we find a strong association between hysterectomy rates and household wealth. Higher education, however, is less influential, a finding borne out by another study in India reporting higher hysterectomy rates among uneducated, rural women[11]. Turning to sterilisation, economic status is not a strong predictor for prevalence but our results indicate that those with at least secondary education are less likely to be

For peer review only - http://bmjopen.bmj.com/site/about/guidelines.xhtml

sterilised. Within groups, even after adjusting for distance and socio-economic factors, CS rates are still higher among non-tribal compared to tribal women, a finding supported by a recent study in western India [25]. Capacity of facilities is associated with higher CS rates but not hysterectomy Hysterectomy is typically a more complex procedure requiring more resources so it is unsurprising that increased capacity in small local facilities does not influence rates.

Interpretation of findings: With respect to distance as a contributing factor for surgical access, travel times to surgical facilities are associated with CS and hysterectomy rates but not sterilisation. The evident lack of association between distance and sterilisation rates may be explained by the greater availability of this procedure at smaller facilities and surgical outreach camps; the 2015-16 DHS reports that in rural areas 53% of sterilisation is provided at a level below a government municipal or main private hospital.[14] Surgery in private facilities where capacity is much more dispersed and dominated by non-poor users is less contingent on travel time. This may be linked to the greater number of smaller private facilities that offer surgery so that travel-time is less important. Rates of CS are substantially above the 10-15% level even for the poorest income groups. These higher rates may result partly from women with more complex deliveries seeking care from these facilities but also an excess preference for CS over non-surgical delivery in private facilities, a concern raised in other studies across India [26].

We find the presence of non-doctor medical staff is generally associated with lower levels of surgical activity. This is supported by other findings: across widely different contexts - for example the US and Iran [27,28] - there is evidence that surgical delivery is lower where more care is provided by nurses and midwives. Our study supports this with lower rates of both CS and sterilisation where similar services can be provided by non-surgical alternatives.

The negative association between membership of the RSBY scheme and CS and lack of association with hysterectomy and CS surgery and the state insurance scheme, also found in other studies, [29] remains puzzling given that both form part of the respective insurance packages. One explanation is that since RSBY is largely a scheme for the poor, the negative effect of lower income over-ride any positive impact of holding insurance. Another explanation is that beneficiaries are unware of benefits of insurance. This was suggested in a recent study of RSBY which revealed that lack of awareness of scheme benefits resulted in RSBY beneficiaries accessing paid, private services rather than through RSBY facilities [30]. A further explanation may be that the reimbursement rates for surgery across the states are lower than the costs of services making facilities less likely to accept insured patients. Delays and other issues with the insurance mechanisms also often make private providers reluctant to participate in schemes. [30] The findings suggest that access to surgery is not

For peer review only - http://bmjopen.bmj.com/site/about/guidelines.xhtml

guaranteed by inclusion in an insurance benefits package. It is also important to ensure that full costs of delivering services are properly compensated particularly for providers that are delivering services to more vulnerable groups.

Limitations: The study has a number of limitations. The self-reported DHS data does not indicate whether surgery was clinically indicated at an individual level so drawing definitive conclusions on excessive surgery at an individual level is therefore not possible. Nevertheless, the population estimates suggest rates that for some groups are too high and some too low. The distance variable is a rough estimate of the actual time to a facility because of the geographic displacement in the GIS variable and the assumption that facilities are based in the centre of the district in the absence of precise GIS coordinates for facilities. Finally, the study takes no account of the quality of service provided in facilities, a variable that in itself is likely to be an important but missing determinant of use of services.

Implications for policy and practice: The huge variation in access to gynaecological and obstetric surgery across North-East India illustrates the persistent importance of factors that both discourage essential surgical care and incentivise some unnecessary surgery. Evidence from a range of contexts suggest that supply-side improvements will not increase use of services without mitigating demand barriers.[31] Cost-effective solutions to alleviate these barriers might either take the form of increasing services in remoter areas (e.g. through outreach services plus rural case finding as practised in other contexts[32]), increasing the ability of vulnerable groups to access geographically more accessible private services at low cost, or attempting to compensate for the greater travel costs of those living in remoter areas. Elsewhere in South Asia, such compensation has been shown to be important in increasing service use.[33,34] Consideration might be given to incorporating such compensation into financing mechanisms.

While increasing access to services is important for some, for others policy needs to focus on limiting excessive surgery. For CS and hysterectomy high rates are present for richer and more educated groups but for sterilisation higher use is evident for those with less education. Policy to reduce unnecessary surgery in rich and poor households requires ensuring adequate and accessible staff to promote and undertake non-surgical alternatives in both rural and urban areas. At the same time, further attention is required to ensure that surgery is accessible when required by mitigating demand-side barriers for those with clear clinical need.

Contributorship statement

For peer review only - http://bmjopen.bmj.com/site/about/guidelines.xhtml

Tim Ensor conceived and designed the study undertook the data analysis, contributed to interpretation of the results and was wrote the first draft of the paper. **Amrit Virk** helped guide the development of study, interpretation of results and contributed to policy conclusions. She read and edited the final draft. **Noel Aruparauil** provided input on the interpretation of results particularly around clinical aspects of health seeking behaviour. He read and edited the final draft.

Competing interests

The authors declare no competing interests

Funding

This research was funded by the National Institute for Health Research (NIHR) (16/137/44) using UK aid from the UK Government to support global health research.

Data sharing statement

The study is based on a secondary analysis of data available in publicly available repositories. The NHFS is available at dhsprogram.com. HMIS data is available at nrhm-mis.nic.in/SitePages/Home.aspx. No additional data generated from this study are available.

Acknowledgement: We acknowledge the helpful comments of three independent reviewers. The views expressed in this publication are those of the author(s) and not necessarily those of the NIHR or the UK Department of Health and Social Care.

For peer review only

References

1. Rose J, Weiser TG, Hider P, Wilson L, Gruen RL, Bickler SW. Estimated need for surgery worldwide based on prevalence of diseases: a modelling strategy for the WHO Global Health Estimate. *The Lancet Global health* 2015; **3 Suppl 2**: S13-20.
2. Alkire BC, Raykar NP, Shrime MG, et al. Global access to surgical care: a modelling study. *The Lancet Global health* 2015; **3**(6): e316-23.
3. Brownlee S, Chalkidou K, Doust J, et al. Evidence for overuse of medical services around the world. *Lancet* 2017; **390**(10090): 156-68.
4. Bae JY, Groen RS, Kushner AL. Surgery as a public health intervention: common misconceptions versus the truth. *Bull World Health Organ* 2011; **89**(6): 394.
5. Okoroh JS, Chia V, Oliver EA, Dharmawardene M, Riviello R. Strengthening Health Systems of Developing Countries: Inclusion of Surgery in Universal Health Coverage. *World J Surg* 2015; **39**(8): 1867-74.
6. Mock CN, Donkor P, Gawande A, et al. Essential surgery: key messages from Disease Control Priorities, 3rd edition. *Lancet* 2015; **385**(9983): 2209-19.
7. Meara JG, Leather AJ, Hagander L, et al. Global Surgery 2030: Evidence and solutions for achieving health, welfare, and economic development. *Surgery* 2015; **158**(1): 3-6.
8. Raykar NP, Yorlets RR, Liu C, et al. A qualitative study exploring contextual challenges to surgical care provision in 21 LMICs. *Lancet* 2015; **385 Suppl 2**: S15.
9. Ensor T, Cooper S. Overcoming barriers to health service access: influencing the demand side. *Health Policy Planning* 2004; **19**(2): 69-79.
10. Grimes CE, Bowman KG, Dodgion CM, Lavy CB. Systematic review of barriers to surgical care in low-income and middle-income countries. *World J Surg* 2011; **35**(5): 941-50.
11. Shekhar C, Paswan B, Singh A. Prevalence, sociodemographic determinants and self-reported reasons for hysterectomy in India. *Reprod Health* 2019; **16**(1): 118.
12. Press Information Bureau. North Eastern Region Vision 2020 (Press Release) Delhi: Government of India; 2019.
13. Debas HT, Donkor P, Gawande A, Jamison D, Kruk M, Mock C. Essential Surgery. Third ed. Washington: World Bank Group; 2015.
14. International Institute for Population Sciences. National Health and Family Survey 2015-16. India, Mubai: IIPS, 2017.
15. Government of India. Health Management Information System. 2019. https://nrhm-mis.nic.in/SitePages/Home.aspx.
16. Government of India. censusindia.gov.in/2011census/dchb/DCHB.html. 20112019).
17. World B. Measuring Living Standards: Household Consumption and Wealth Indices, Quantitative Techniques for Health Equity Analysis-Technical Note #4. 2004. http://siteresources.worldbank.org/INTPAH/Resources/Publications/Quantitative-Techniques/health_eq_tn04.pdf
18. Weber S. A simple command to calculate travel distance and travel time. *The State Journal* 2018; **17**(4): 962-71.
19. Saikia D, Kangkana Das K. Rural Health Infratsutures in the North-East India. Munich: Munich Personal RePEc Archive, 2012.
20. Guilmoto CZ, Dumont A. Trends, Regional Variations, and Socioeconomic Disparities in Cesarean Births in India, 2010-2016. *JAMA Netw Open* 2019; **2**(3): e190526.
21. ICF. The DHS Program STATcompiler 2012. www.statcompiler.com. (accessed 15/1/20 2020).
22. Prusty RK, Choithani C, Gupta SD. Predictors of hysterectomy among married women 15-49 years in India. *Reprod Health* 2018; **15**(1): 3.
23. Neuman M, Alcock G, Azad K, et al. Prevalence and determinants of caesarean section in private and public health facilities in underserved South Asian communities: cross-sectional analysis of data from Bangladesh, India and Nepal. *BMJ open* 2014; **4**(12): e005982.

For peer review only - http://bmjopen.bmj.com/site/about/guidelines.xhtml

24. Boatin AA, Schlotheuber A, Betran AP, et al. Within country inequalities in caesarean section rates: observational study of 72 low and middle income countries. *BMJ* 2018; **360**: k55.
25. Desai G, Anand A, Modi D, et al. Rates, indications, and outcomes of caesarean section deliveries: A comparison of tribal and non-tribal women in Gujarat, India. *PLoS One* 2017; **12**(12): e0189260.
26. Singh P, Hashmi G, Swain PK. High prevalence of cesarean section births in private sector health facilities- analysis of district level household survey-4 (DLHS-4) of India. *BMC Public Health* 2018; **18**(1): 613.
27. Kashanian M, Javadi F, Haghighi MM. Effect of continuous support during labor on duration of labor and rate of cesarean delivery. *Int J Gynaecol Obstet* 2010; **109**(3): 198-200.
28. Lydon-Rochelle M. Cesarean delivery rates in women cared for by certified nurse-midwives in the United States: a review. *Birth* 1995; **22**(4): 211-9.
29. Desai S, Shuka A, Nambiar D, Ved R. Patterns of hysterectomy in India: a national and state-level analysis of the Fourth National Family Health Survey (2015-2016). *BJOG* 2019; **126 Suppl 4**: 72-80.
30. Yadav V, Kumar S, Balasubramaniam S, et al. Facilitators and barriers to participation of private sector health facilities in government-led schemes for maternity services in India: a qualitative study. *BMJ open* 2017; **7**(6): e017092.
31. O'Donnell O. Access to health care in developing countries: breaking down demand side barriers. *Cad Saude Publica* 2007; **23**: 2820 - 34.
32. White MC, Hamer M, Biddell J, et al. Facilitating access to surgical care through a decentralised case-finding strategy: experience in Madagascar. *BMJ Glob Health* 2017; **2**(3): e000427.
33. Ensor T, Bhatt H, Tiwari S. Incentivizing universal safe delivery in Nepal: 10 years of experience. *Health Policy Plan* 2017; **32**(8): 1185-92.
34. De Costa A, Patil R, Kushwah SS, Diwan VK. Financial incentives to influence maternal mortality in a low-income setting: making available 'money to transport' - experiences from Amarpatan, India. *Glob Health Action* 2009; **2**.

For peer review only

For peer review only - http://bmjopen.bmj.com/site/about/guidelines.xhtml

Tables

Table 1: Correlation between surgery, income per capita and supply side variables across all states of India

	Surgical procedures per 1,000 population	C-section rate (% live births)	Hysterectomy per 1,000 women	Sterilisations per 1,000 women
All India	3.1	17%	0.8	2.5
NE India States	2.8	18%	0.5	0.8
**R-squared statistics**				
Registered doctors per 1,000	0.373***	0.612***	0.179**	0.003
Government doctors per 1,000	0.281**	0.234**	0.102*	0.252**
Beds per 1,000	0.288**	0.336**	0.090*	0.094*
GDP per capita	0.103*	0.280**	0.047	0.020

Note: significant at the *** 1% level, ** 5% level, * 10% level.

For peer review only

Table 2: Association between use of surgical services and individual, household and supply side characteristics (Multivariate logit with cluster adjusted standard errors)

	Caesarean Section			Hysterectomy			Sterilisation		
	Coef.	t-statistic	sig.	Coef.	t-statistic	sig.	Coef.	t-statistic	sig.
**Wealth group (base=poorest)**									
poorer	0.483	3.840	***	0.495	2.480	**	0.013	0.190	
middle	1.093	8.390	***	0.739	3.610	***	0.105	1.380	
richer	1.629	11.660	***	1.162	5.480	***	0.184	2.010	**
richest	2.072	13.340	***	1.330	5.480	***	0.459	3.740	***
	-	-		-	-		-	-	
**Education (base=no education)**	-	-		-	-		-	-	
Completed primary	0.552	3.660	***	0.301	1.900	*	- 0.012	- 0.170	
Completed secondary	0.834	6.370	***	- 0.080	- 0.590		- 0.382	- 5.460	***
Completed Higher	1.230	7.950	***	- 0.253	- 0.980		- 0.931	- 7.100	***
	-	-		-	-		-	-	
**Parity (base=1 delivery)**	-	-		-	-		-	-	
	-	-		-	-		-	-	
Two	- 0.641	- 9.060	***	0.136	0.780		2.432	18.630	***
Three	- 1.291	- 12.190	***	0.162	0.880		2.914	21.090	***
Four or more	- 1.882	- 13.170	***	- 0.145	- 0.780		2.823	19.450	***
	-	-		-	-		-	-	
**Marital status (base=never married)**	-	-		-	-		-	-	
married	- 0.763	- 1.380		1.074	2.400	**	1.078	1.350	
widowed	- 0.875	- 1.360		0.579	1.180		0.424	0.530	
divorced	- 0.694	- 1.050		1.233	2.140	**	0.473	0.560	
living together/separated	- 0.999	- 1.530		0.108	0.170		0.619	0.730	
	-	-		-	-		-	-	
**Religion (base=hindu)**	-	-		-	-		-	-	
muslim	- 0.660	- 5.840	***	- 0.723	- 3.230	***	- 1.649	- 14.340	***
christian	- 0.281	- 2.510	**	- 0.014	- 0.070		- 0.298	- 2.850	***
sikh	3.705	3.090	***	-	-		0.273	0.200	
buddhist/neo-buddhist	- 0.671	- 3.850	***	0.347	0.850		- 0.225	- 1.350	
jain	- 1.851	- 1.320		3.264	2.670	***	1.336	2.190	**
no religion	0.436	0.740		- 0.086	- 0.110		- 1.007	- 2.540	**
other	- 0.111	- 0.860		0.230	1.020		- 1.077	- 9.420	***
	-	-		-	-		-	-	

For peer review only

For peer review only - http://bmjopen.bmj.com/site/about/guidelines.xhtml

	Caesarean Section			Hysterectomy			Sterilisation		
	Coef.	t-statistic	sig.	Coef.	t-statistic	sig.	Coef.	t-statistic	sig.
**Caste/Tribe (base=none)**	-	-		-	-		-	-	
Caste	- 0.151	- 1.360		- 0.100	- 0.530		0.165	1.620	
Tribe	- 0.562	- 4.100	***	- 0.259	- 0.970		- 0.148	- 1.150	
	-	-		-	-		-	-	
**Age group (base=15-19)**	-	-		-	-		-	-	
20-25	0.589	3.460	***	- 2.426	- 3.740	***	0.869	1.190	
25-30	0.913	5.260	***	- 1.229	- 4.290	***	1.289	1.800	*
30-35	1.311	7.190	***	0.103	0.510		1.671	2.320	**
35-40	1.589	8.340	***	0.821	5.430	***	1.810	2.500	**
40-45	1.613	6.630	***	0.529	4.360	***	1.715	2.380	**
45-50	1.371	2.550	**	-	-		1.607	2.220	**
								-	
**Type of insurance (base=no insurance)**								-	
state	0.072	0.580		0.017	0.120		0.226	2.550	**
rsby	- 0.254	- 2.170	**	0.092	0.470		0.266	3.550	***
Other insurance	0.252	1.100		0.008	0.020		0.434	2.800	***
	-	-		-	-		-	-	
**Supply-side variables**	-	-		-	-		-	-	
Drs/1,000 population	0.022	1.670	*	- 0.043	- 1.670	*	0.031	3.090	***
Other medical/1,000 population	- 0.021	- 2.930	***	0.011	0.990		- 0.044	- 7.050	***
Time to main surgical facility	- 0.001	- 3.550	***	- 0.001	- 2.340	**	- 0.000	- 0.710	
	-	-		-	-		-	-	
Beds/1,000 population	0.017	3.690	***	0.005	0.720		0.025	6.620	***
	-	-		-	-		-	-	
Constant	- 2.494	- 4.120	***	-	-		- 6.695	- 6.320	***

Note: Significant at the *** 1% level, ** 5% level, * 10% level.

For peer review only

For peer review only - http://bmjopen.bmj.com/site/about/guidelines.xhtml

Table 3: costs of delivery care (US dollars and % of per capita income)

	Public	Private	Total	Public	Private	Total
**Non-surgical delivery**	Cost per delivery			% of per capita income		
Facility	$ 38	$ 140	$ 50	3.3%	12.0%	4.3%
Transport	$ 5	$ 9	$ 6	0.4%	0.8%	0.5%
Total	$ 44	$ 148	$ 56	3.8%	12.8%	4.8%
**Surgical delivery**						
Facility	$ 120	$ 311	$ 199	10.3%	26.8%	17.1%
Transport	$ 8	$ 15	$ 11	0.7%	1.3%	0.9%
Total	$ 128	$ 325	$ 210	11.0%	28.0%	18.1%

Note: state-wise GDP obtained from http://mospi.nic.in/data

Figure legends

Figure 1: Major surgeries per 1,000 population across states of India (2016-17)

Figure 2: Proportion of women that reported undergoing three gynaecological/obstetric procedures

Figure 3: Variation in CS by distance from substantial surgical facility

Figure 4: Variation in hysterectomy (prevalence) by distance from substantial surgical facility

Figure 5: Caesarean section rate as proportion of all deliveries in public and private facilities

For peer review only

For peer review only - http://bmjopen.bmj.com/site/about/guidelines.xhtml

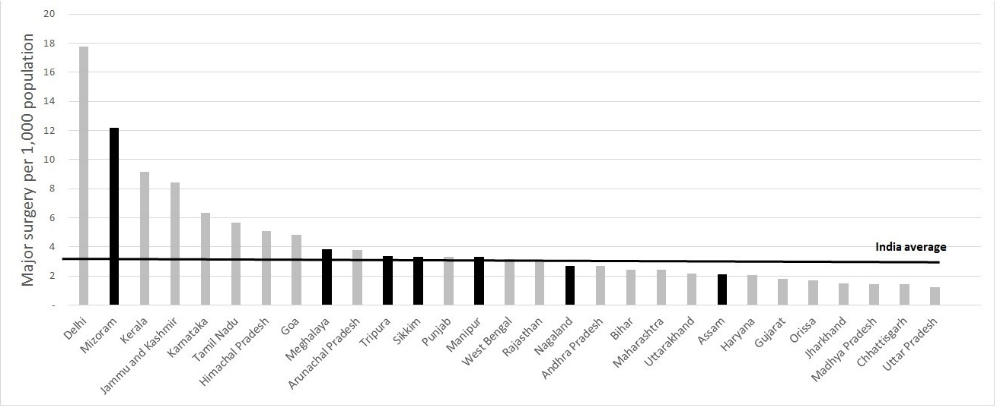

Figure 1: Major surgeries per 1,000 population across states of India (2016-17)

278x115mm (120 x 120 DPI)

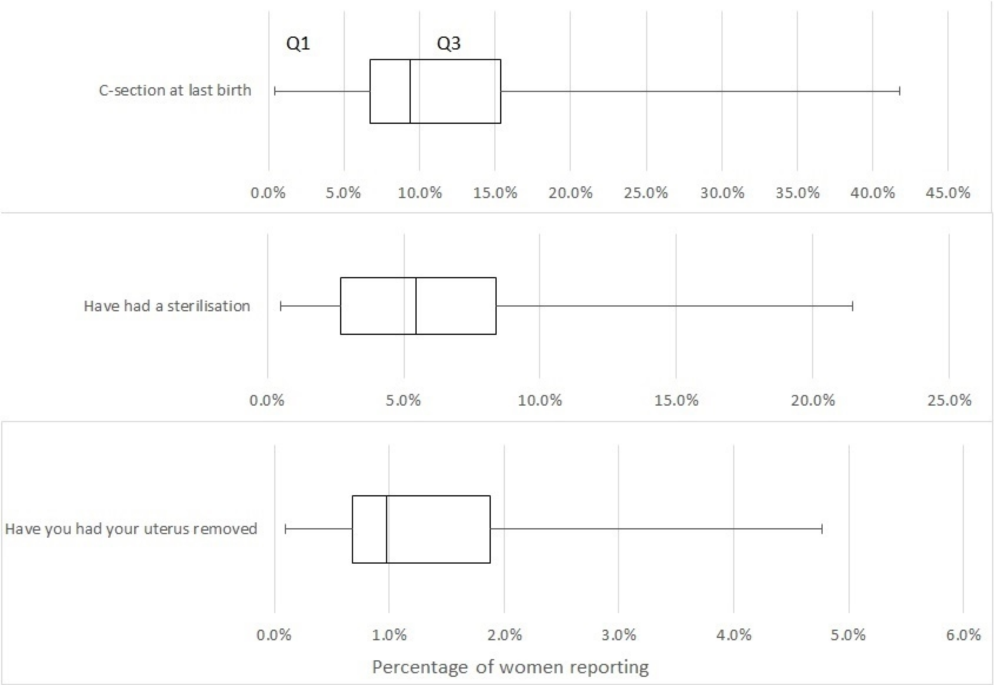

Figure 2: Proportion of women that reported undergoing three gynaecological/obstetric procedures

179x124mm (120 x 120 DPI)

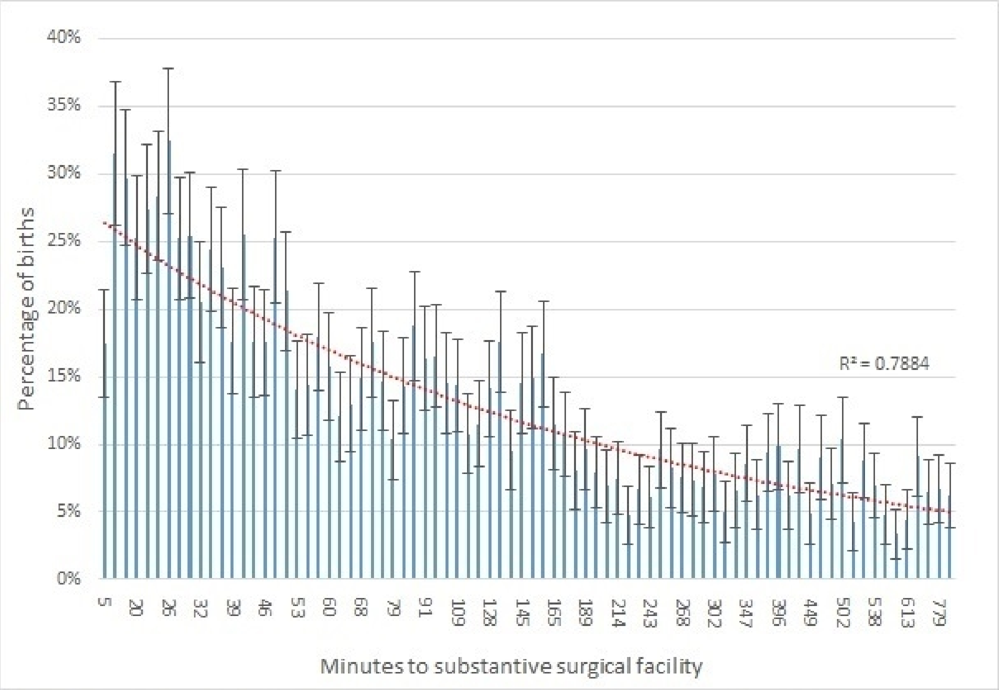

Figure 3: Variation in CS by distance from substantial surgical facility

128x89mm (120 x 120 DPI)

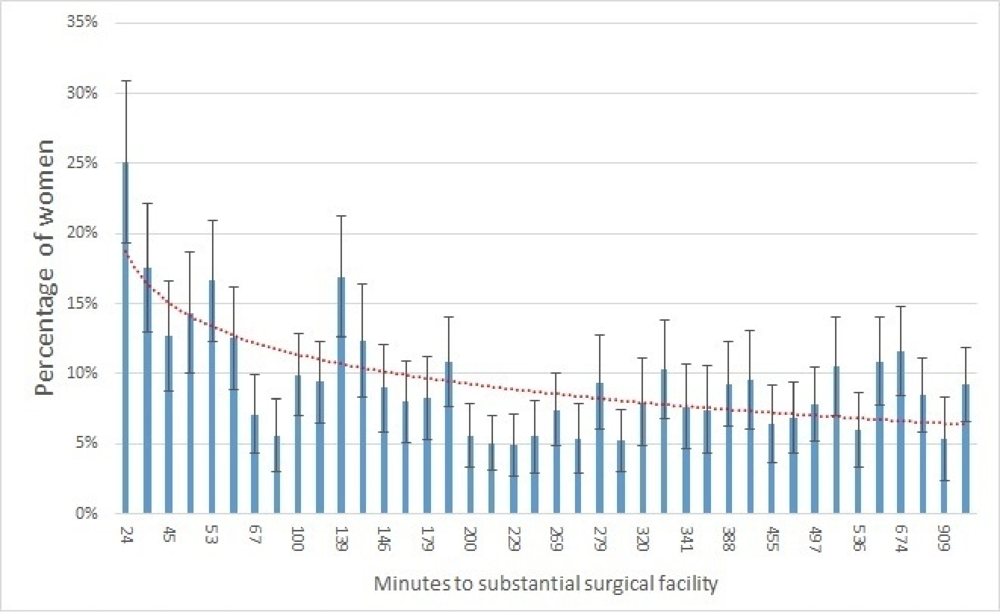

Figure 4: Variation in hysterectomy (prevalence) by distance from substantial surgical facility

139x85mm (120 x 120 DPI)

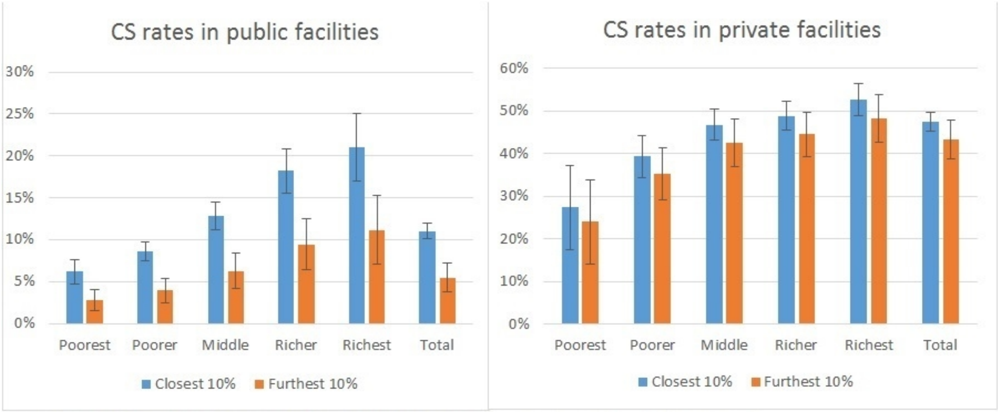

Figure 5: Caesarean section rate as proportion of all deliveries in public and private facilities

190x78mm (120 x 120 DPI)

BMJ Open

Factors influencing use of essential surgical services in North-East India: a cross sectional study of obstetric and gynaecological surgery

Journal:	*BMJ Open*
Manuscript ID	bmjopen-2020-038470.R2
Article Type:	Original research
Date Submitted by the Author:	28-Sep-2020

Complete List of Authors:	Ensor, Tim; University of Leeds, Nuffield Centre for Health<br>Virk, Amrit; University of Leeds<br>Aruparayil, Noel; University of Leeds

**Primary Subject Heading**:	Global health
Secondary Subject Heading:	Health policy, Public health
Keywords:	Maternal medicine < OBSTETRICS, Health policy < HEALTH SERVICES ADMINISTRATION & MANAGEMENT, Adult surgery < SURGERY

SCHOLARONE™
Manuscripts

I, the Submitting Author has the right to grant and does grant on behalf of all authors of the Work (as defined in the below author licence), an exclusive licence and/or a non-exclusive licence for contributions from authors who are: i) UK Crown employees; ii) where BMJ has agreed a CC-BY licence shall apply, and/or iii) in accordance with the terms applicable for US Federal Government officers or employees acting as part of their official duties; on a worldwide, perpetual, irrevocable, royalty-free basis to BMJ Publishing Group Ltd ("BMJ") its licensees and where the relevant Journal is co-owned by BMJ to the co-owners of the Journal, to publish the Work in this journal and any other BMJ products and to exploit all rights, as set out in our licence.

The Submitting Author accepts and understands that any supply made under these terms is made by BMJ to the Submitting Author unless you are acting as an employee on behalf of your employer or a postgraduate student of an affiliated institution which is paying any applicable article publishing charge ("APC") for Open Access articles. Where the Submitting Author wishes to make the Work available on an Open Access basis (and intends to pay the relevant APC), the terms of reuse of such Open Access shall be governed by a Creative Commons licence – details of these licences and which Creative Commons licence will apply to this Work are set out in our licence referred to above.

Other than as permitted in any relevant BMJ Author's Self Archiving Policies, I confirm this Work has not been accepted for publication elsewhere, is not being considered for publication elsewhere and does not duplicate material already published. I confirm all authors consent to publication of this Work and authorise the granting of this licence.

For peer review only

Title: Factors influencing use of essential surgical services in North-East India: a cross sectional study of obstetric and gynaecological surgery.

Authors:

Tim Ensor, PhD, University of Leeds, University of Leeds, Worsley Building, Clarendon Way, Leeds, LS2 9NL, + 44 (0) 113 343 6908, t.r.a.ensor@leeds.ac.uk, ORCID ID 0000-0003-0279-9576, (corresponding author)

Amrit Virk, PhD, University of Leeds, A.Virk@leeds.ac.uk, ORICD ID 0000-0001-8686-2776

Noel Aruparayil, MBBS, University of Leeds, N.K.Aruparayil@leeds.ac.uk, ORCID ID 0000-0002-2898-772X

-

For peer review only - http://bmjopen.bmj.com/site/about/guidelines.xhtml

Abstract: (250 words)

Introduction: There continues to be a large gap between need and actual use of surgery in low-resource settings. While policy frequently focuses on expanding the supply of services, demand-side factors are at least as important in determining under and over utilisation. The aim of this study is to understand how these factors influence the use of selected essential obstetric and gynaecological surgical procedures in the underserved and remote setting of North-East India.

Methods: The study combines and makes use of data from a variety of surveys and routine systems. Descriptive analysis of variations in caesarean section, hysterectomy and sterilisation and then multivariate logit analysis of demand and supply-side factors on access to these services is undertaken.

Results: Surgical rates vary substantially both across and within North-East India, correlated with service capacity and socio-economic status. Travel times to surgical facilities are associated with rates of caesarean section and hysterectomy but not sterilisation where services are much more deconcentrated. Travel is less important for surgery in private facilities where capacity is much more dispersed but dominated by the non-poor. The presence of non-doctor medical staff is associated with lower levels of surgical activity.

Conclusion: In low resource, remote settings policy interventions to improve access to services must recognise that surgical rates in low-resource settings are heavily influenced by demand-side factors. As well as boosting services, mechanisms need to mitigate demand-side barriers particularly distance and influence practice to encourage surgical intervention only where clinically indicated.

Key words: surgery; obstetrics and gynaecology; maternal health; demand-side barriers; distance

For peer review only

For peer review only - http://bmjopen.bmj.com/site/about/guidelines.xhtml

Article summary

Strengths and limitations of this study

- Combines three large data sets on obstetric services to allow an exploration of the supply and demand factors affecting use of obstetric and gynaecological services.
- Makes use of a distance variable that allows consideration of the effect of location on demand for services
- The distance variable is a rough estimate of the actual time to a facility because of the geographic displacement in the GIS variable and the assumption that facilities are based in the centre of the district in the absence of precise GIS coordinates for facilities.
- DHS data does not indicate whether surgery was clinically indicated at an individual level so drawing definitive conclusions on excessive surgery at an individual level is therefore not possible.
- Relies on self-reported use of surgical services.

For peer review only

For peer review only - http://bmjopen.bmj.com/site/about/guidelines.xhtml

Introduction

There continues to be a huge difference between user need and use of surgical care in low-resource settings with up to 4.8 billion lacking accessible services.[1,2] Use of services is typified by substantial inequality with both under and over-use of surgical services harming human health[3]. It is estimated that increased appropriate access to surgical services could have a substantial impact on the global burden of disease.[4] Improved surgery is a key component in strengthening a system to deliver universal health coverage.[5,6]

Much recent focus on access to global surgery has been on strengthening the supply side through increasing surgical staff, improving training and infrastructure provision [7,8]. A strong supply-side is necessary but it is not sufficient for improved access. A host of demand-side factors at the user level such as affordability, proximity to services, socioeconomics and education influence service use.[9] Understanding how these factors influence surgical access is crucial for the design of evidence-based policy interventions yet there is limited evidence on the size of their impact particularly in low resource settings. [2,10,11]

This study aims to examine the extent to which demand and supply factors influence surgical use in such underserved, poorly resourced and remote settings. Our focus is on the eight states in North-East India, one of the most underdeveloped regions in the country with persistently poor health outcomes and substantial access barriers.[12] We examine factors determining use of three essential surgical procedures: caesarean sections, hysterectomy and female sterilisation [13]. Although some of these factors are likely to be specific to this type of surgery others can be regarded as more general. In this respect, these procedures act as tracer services to identify general issues of access to surgical provision.

Methods

Data sources: This cross sectional study based on secondary data sources makes use of three data sets. The National Family Health Survey (NFHS) 2015-16 provides a sample of 98,700 women of reproductive age that is statistically representative down to district level[14]. The online HMIS provides information on surgical activity and was used to identify centres with surgical capability.[15] The District Census provides supply-side information on numbers of facilities, staff, beds and population by district.[16]

Variables and method of analysis: Descriptive statistics on state and district level surgical activity in North-East India are compared with other states and presented to set the regional discussion in context. Subsequently, for the eight North-Eastern states (Arunachal Pradesh, Assam, Manipur,

For peer review only

Meghalaya, Mizoram, Nagaland, Sikkim and Tripura) multivariate logit analysis is used to examine the association between use by women of three surgical procedures, individual and household characteristics (age, education, parity, caste/tribe, marital status, insurance held and economic status), proximity to and capacity of health services. The p-values for the key model coefficients were estimated based on the t-statistic with 95% significance used as the threshold for valid inference. The stata margins command is used to estimate the relationship between the outcome and individual factors holding other independent variables at their mean values.[17] The economic status of the household is approximated by the asset-based wealth index.[18] Three supply variables are used, available at the village level from the district-level census: doctors, other medical staff and beds expressed as rate per 1,000 population.

The distance between clusters of households and main surgical centres is computed through a two stage process. The NFHS provides approximate geographical positioning of the cluster from which households are selected. In line with the procedure adopted across DHS surveys, coordinates are then shifted randomly by up to five kilometres in rural and two kilometres in urban areas to protect the anonymity of responding households. HMIS data provides information on the amount of surgical activity in facilities within a district allowing computation of the size of their surgical capacity defined as high (more than 2000 major surgical procedures a year) through to low (less than one per day). The time it takes to travel from the centre of each district to all other districts with high surgical activity is calculated in Stata using the georoutei add-in.[19] This takes pairs of coordinates (longitude and latitude) and accesses the HERE mapping database (developer.here.com) to compute the total travel time using available roads adjusted for speed of traffic. We calculated the distance between each district centre and the nearest district with surgical capacity which was then added to the distance between each household cluster and district centre, producing an estimate of total time to a surgical centre.

Ethical considerations: The data used in this study are all anonymised and in the public domain and no additional ethics approval was required in order to undertake the study. DHS was downloaded from the Measure website (dhsprogram.com) while district HMIS and census data are available through public facing websites (censusindia.gov.in; nrhm-mis.nic.in). The funder of the research had no role in the design of the surveys used (NFHS, HMIS or District Census) nor any role in the , analysis and interpretation of data, writing the report or the decision to submit the paper for publication. Patients or the public were not involved in the design, conduct, reporting or dissemination plans of our research

For peer review only - http://bmjopen.bmj.com/site/about/guidelines.xhtml

Results

Nationally, the HMIS reports a total of 4.3 million (3.1 per 1,000 population) major (with spinal/general anaesthesia) and 11.5 million (8.5 per 1,000 population) minor surgical procedures in 2017-18. The rates vary from 1.2/1,000 major surgical procedures in the country's most populous state Uttar Pradesh to 18/1000 in largely urban city-state Delhi. Rates are much lower than the needs based benchmark of 45/1,000 major surgical procedures for South Asia.[1] With the exception of Mizoram state, where health service infrastructure appears to be better functioning than in other states,[20] rates of surgery across the North East are at or below the country average of 3.1 per 1,000 population (Figure 1).

<Figure 1 about here>

The national caesarean section (CS) rate is reported by place of service focused HMIS as 17.5% of live births for 2017-18 while the household focused NFHS reports a similar figure.[14] This average, which is higher than the WHO benchmark for necessary surgical delivery of 10-15% [21], masks substantial state-wide variation ranging from 2.5% in Bihar to over 45% in Tamil Nadu.[15] The NFHS reported 31% of Indian women that had a sterilisation which is close to the highest rate internationally—33% in the Dominican Republic.[22] Comparing hysterectomy rates across countries is impeded by the nature of the survey data. The NFHS focuses on women of reproductive age where as in established health systems hysterectomy is largely concentrated in older age groups. Studies in India have drawn attention to the relatively high rates of the procedure with one third occurring in women below the age of 40.[23] In NE India rates of gynaecological and obstetric surgery are at or below the national average: CS rates range from 7.9% (Meghalaya) to 21.8% (Tripura) of deliveries (national average=17.5%), hysterectomy from 0.2 (Assam) to 2.8 (Arunachal Pradesh) per 1,000 women (national average=0.8)) and sterilisation 0.2 (Manipur) to 1 (Assam) per 1,000 women (national average=2.6).

<Table 1 about here>

Turning to surgical utilisation, across India use of surgery (per 1,000 population) is positively associated both with the capacity of health services and the wealth of individuals (Table 1) with associations strongest for CS. The stronger association with the total number of registered doctors (per 1,000 population) in the state rather than number of government doctors (per 1,000 population) may reflect the importance of the private sector in delivering the majority of CS across the country. According to the HMIS, 51% of CS were delivered in private facilities in 2016-17 while the NFHS reports 53% for births in 2014-2016. The association with health service capacity and individual wealth is much weaker for hysterectomy and sterilisations. Sterilisation (per 1,000

For peer review only

women) is positively associated with the total number of government doctors but not total registered doctors in states, indicating public sector dominance over the provision of permanent contraception methods with 74% provided in the public sector.[15]

Across NE India, use of surgical services varies widely: CS as a proportion of deliveries by district varies from 0.4% to almost 42% (Interquartile range – IQR - 8.7%), hysterectomy from 0.1% to 4.8% (IQR 1.2%). Rates of female sterilisation vary from 0.5% to more than 21% (IQR 5.7%) (Figure 2).

<Figure 2 about here>

Surgical use is positively associated with proximity to major surgical centres. CS rates, for example, range from over 25% for clusters than are geographically close to major surgical centres to 5% or lower in areas more than ten hours away (Figure 3). There is a less pronounced relationship between distance and prevalence of hysterectomy (Figure 4) while no clear association is found for sterilisation.

<Figure 3 about here>

<Figure 4 about here>

Turning to the analysis of individual behaviour, all three surgical procedures are positively associated with household wealth (Table 2). This association is strongest for CS and weakest for sterilisation. CS rates are substantially higher for women who are better educated. In contrast, hysterectomy and sterilisation rates are lower for those with secondary education or above compared to those without any education.

<Table 2 about here>

The importance of economic status is likely to be partly the result of cost barriers to obtaining care. Costs of surgical delivery, including transport represent a substantial proportion of per capita income; 11% in the public sector and 28% in the private sector (Table 3). The NFHS found that after distance (36%), treatment cost was the second most important factor (21%) for women not delivering in a facility. In contrast, sterilization costs were reportedly less burdensome for users as 75% of sterilized women in the NFHS reported not paying anything and 48% reported receiving financial compensation.[14]

<Table 3 about here>

For peer review only - http://bmjopen.bmj.com/site/about/guidelines.xhtml

The incidence of caesarean section increases with age particularly for those over 30 compared to younger women. The prevalence of hysterectomy and sterilisation is also positively associated with age the latter likely reflecting an increased demand for birth control as family size grows. Higher parity is also associated with greater prevalence of sterilisation. The analysis suggests that women with Muslim or Christian faith are less likely to undergo the three procedures while those living in tribal communities have lower levels of CS than other communities. There is no association with the other gynaecological surgery.

RSBY (Rashtriya Swasthya Bima Yojana) insurance is the most common insurance in the NE states with the exception of Arunachal Pradesh and Sikkim where state insurance dominates. RSBY membership is negatively associated with caesarean section and has no statistically significant association with hysterectomy. Sterilisation is positively associated with RSBY as well as other types of insurance (Table 2).

Distance to a major surgical centre is negatively associated with both caesarean section and hysterectomy but no statistically significant relationship is found for sterilisation. No information on where sterilisation was carried out is available in the NFHS and those reporting place of hysterectomy is too small for a meaningful analysis. The CS rates vary substantially across the public and private sector for all income groups. Overall CS rates exceed 40% for women delivering in private facilities falling to 11% in public facilities. There is a strongly pronounced income gradient for both facility types (Figure 5).

Proximity to medical services, as measured by doctor numbers, is shown to be positively associated with CS and hysterectomy rates. Availability of more local doctors may also increase referral to facilities even if not situated in the area. Local bed capacity is associated with CS rates but not with hysterectomy. In fact across the eight states, provision of hysterectomy is highly concentrated with just 12% of districts carrying out almost 70% of hysterectomies in 2017/19 (HMIS).

<Figure 4 about here>

The association with distance from a main surgical facility is notably different in public and private facilities. For public facilities there is a large and statistically significant difference between women living close versus those living far from a large surgical facility (Figure 5). In contrast, for women delivering in private facilities, the effect of proximity is not nearly so pronounced and not significant for any income group.

For peer review only - http://bmjopen.bmj.com/site/about/guidelines.xhtml

Discussion

Key findings: Surgical rates vary substantially both across and within the states of North-East India, correlated with service capacity and socio-economic status. The results demonstrate the importance of individual level demand-side factors on the use of surgical services across NE India. The strong positive relationship between economic and educational status and CS is evidenced in other studies in South Asia and across LMICs. [24,25]. Like recent studies on hysterectomy in India[23], we find a strong association between hysterectomy rates and household wealth. Higher education, however, is less influential, a finding borne out by another study in India reporting higher hysterectomy rates among uneducated, rural women[11]. Turning to sterilisation, economic status is not a strong predictor for prevalence but our results indicate that those with at least secondary education are less likely to be sterilised. Within groups, even after adjusting for distance and socio-economic factors, CS rates are still higher among non-tribal compared to tribal women, a finding supported by a recent study in western India [26]. Capacity of facilities is associated with higher CS rates but not hysterectomy Hysterectomy is typically a more complex procedure requiring more resources so it is unsurprising that increased capacity in small local facilities does not influence rates.

Interpretation of findings: With respect to distance as a contributing factor for surgical access, travel times to surgical facilities are associated with CS and hysterectomy rates but not sterilisation. The evident lack of association between distance and sterilisation rates may be explained by the greater availability of this procedure at smaller facilities and surgical outreach camps; the 2015-16 DHS reports that in rural areas 53% of sterilisation is provided at a level below a government municipal or main private hospital.[14] Surgery in private facilities where capacity is much more dispersed and dominated by non-poor users is less contingent on travel time. This may be linked to the greater number of smaller private facilities that offer surgery so that travel-time is less important. Rates of CS are substantially above the 10-15% level even for the poorest income groups. These higher rates may result partly from women with more complex deliveries seeking care from these facilities but also an excess preference for CS over non-surgical delivery in private facilities, a concern raised in other studies across India [27].

We find the presence of non-doctor medical staff is generally associated with lower levels of surgical activity. This is supported by other findings: across widely different contexts - for example the US and Iran [28,29] - there is evidence that surgical delivery is lower where more care is provided by nurses and midwives. Our study supports this with lower rates of both CS and sterilisation where similar services can be provided by non-surgical alternatives.

For peer review only - http://bmjopen.bmj.com/site/about/guidelines.xhtml

The negative association between membership of the RSBY scheme and CS and lack of association with hysterectomy and CS surgery and the state insurance scheme, also found in other studies, [30] remains puzzling given that both form part of the respective insurance packages. One explanation is that since RSBY is largely a scheme for the poor, the negative effect of lower income over-ride any positive impact of holding insurance. Another explanation is that beneficiaries are unware of benefits of insurance. This was suggested in a recent study of RSBY which revealed that lack of awareness of scheme benefits resulted in RSBY beneficiaries accessing paid, private services rather than through RSBY facilities [31]. A further explanation may be that the reimbursement rates for surgery across the states are lower than the costs of services making facilities less likely to accept insured patients. Delays and other issues with the insurance mechanisms also often make private providers reluctant to participate in schemes. [31] The findings suggest that access to surgery is not guaranteed by inclusion in an insurance benefits package. It is also important to ensure that full costs of delivering services are properly compensated particularly for providers that are delivering services to more vulnerable groups.

Limitations: The study has a number of limitations. The self-reported DHS data does not indicate whether surgery was clinically indicated at an individual level so drawing definitive conclusions on excessive surgery at an individual level is therefore not possible. Nevertheless, the population estimates suggest rates that for some groups are too high and some too low. The distance variable is a rough estimate of the actual time to a facility because of the geographic displacement in the GIS variable and the assumption that facilities are based in the centre of the district in the absence of precise GIS coordinates for facilities. Finally, the study takes no account of the quality of service provided in facilities, a variable that in itself is likely to be an important but missing determinant of use of services.

Implications for policy and practice: The huge variation in access to gynaecological and obstetric surgery across North-East India illustrates the persistent importance of factors that both discourage essential surgical care and incentivise some unnecessary surgery. Evidence from a range of contexts suggest that supply-side improvements will not increase use of services without mitigating demand barriers.[32] Cost-effective solutions to alleviate these barriers might either take the form of increasing services in remoter areas (e.g. through outreach services plus rural case finding as practised in other contexts[33]), increasing the ability of vulnerable groups to access geographically more accessible private services at low cost, or attempting to compensate for the greater travel costs of those living in remoter areas. Elsewhere in South Asia, such compensation has been shown to be important in increasing service use.[34,35] Consideration might be given to incorporating such compensation into financing mechanisms.

For peer review only - http://bmjopen.bmj.com/site/about/guidelines.xhtml

For peer review only

While increasing access to services is important for some, for others policy needs to focus on limiting excessive surgery. For CS and hysterectomy high rates are present for richer and more educated groups but for sterilisation higher use is evident for those with less education. Policy to reduce unnecessary surgery in rich and poor households requires ensuring adequate and accessible staff to promote and undertake non-surgical alternatives in both rural and urban areas. At the same time, further attention is required to ensure that surgery is accessible when required by mitigating demand-side barriers for those with clear clinical need.

Contributorship statement

Tim Ensor conceived and designed the study undertook the data analysis, contributed to interpretation of the results and was wrote the first draft of the paper. **Amrit Virk** helped guide the development of study, interpretation of results and contributed to policy conclusions. She read and edited the final draft. **Noel Aruparayil** provided input on the interpretation of results particularly around clinical aspects of health seeking behaviour. He read and edited the final draft.

Competing interests

The authors declare no competing interests

Funding statement

This research was funded by the National Institute for Health Research (NIHR) (16/137/44) using UK aid from the UK Government to support global health research.

Data sharing statement

The study is based on a secondary analysis of data available in publicly available repositories. The NHFS is available at dhsprogram.com. HMIS data is available at nrhm-mis.nic.in/SitePages/Home.aspx. No additional data generated from this study are available.

Acknowledgement: We acknowledge the helpful comments of three independent reviewers. The views expressed in this publication are those of the author(s) and not necessarily those of the NIHR or the UK Department of Health and Social Care.

For peer review only - http://bmjopen.bmj.com/site/about/guidelines.xhtml

For peer review only

References

1. Rose J, Weiser TG, Hider P, Wilson L, Gruen RL, Bickler SW. Estimated need for surgery worldwide based on prevalence of diseases: a modelling strategy for the WHO Global Health Estimate. *The Lancet Global health* 2015; **3 Suppl 2**: S13-20.
2. Alkire BC, Raykar NP, Shrime MG, et al. Global access to surgical care: a modelling study. *The Lancet Global health* 2015; **3**(6): e316-23.
3. Brownlee S, Chalkidou K, Doust J, et al. Evidence for overuse of medical services around the world. *Lancet* 2017; **390**(10090): 156-68.
4. Bae JY, Groen RS, Kushner AL. Surgery as a public health intervention: common misconceptions versus the truth. *Bull World Health Organ* 2011; **89**(6): 394.
5. Okoroh JS, Chia V, Oliver EA, Dharmawardene M, Riviello R. Strengthening Health Systems of Developing Countries: Inclusion of Surgery in Universal Health Coverage. *World J Surg* 2015; **39**(8): 1867-74.
6. Mock CN, Donkor P, Gawande A, et al. Essential surgery: key messages from Disease Control Priorities, 3rd edition. *Lancet* 2015; **385**(9983): 2209-19.
7. Meara JG, Leather AJ, Hagander L, et al. Global Surgery 2030: Evidence and solutions for achieving health, welfare, and economic development. *Surgery* 2015; **158**(1): 3-6.
8. Raykar NP, Yorlets RR, Liu C, et al. A qualitative study exploring contextual challenges to surgical care provision in 21 LMICs. *Lancet* 2015; **385 Suppl 2**: S15.
9. Ensor T, Cooper S. Overcoming barriers to health service access: influencing the demand side. *Health Policy Planning* 2004; **19**(2): 69-79.
10. Grimes CE, Bowman KG, Dodgion CM, Lavy CB. Systematic review of barriers to surgical care in low-income and middle-income countries. *World J Surg* 2011; **35**(5): 941-50.
11. Shekhar C, Paswan B, Singh A. Prevalence, sociodemographic determinants and self-reported reasons for hysterectomy in India. *Reprod Health* 2019; **16**(1): 118.
12. Press Information Bureau. North Eastern Region Vision 2020 (Press Release) Delhi: Government of India; 2019.
13. Debas HT, Donkor P, Gawande A, Jamison D, Kruk M, Mock C. Essential Surgery. Third ed. Washington: World Bank Group; 2015.
14. International Institute for Population Sciences. National Health and Family Survey 2015-16. India, Mubai: IIPS, 2017.
15. Government of India. Health Management Information System. 2019. https://nrhm-mis.nic.in/SitePages/Home.aspx.
16. Government of India. censusindia.gov.in/2011census/dchb/DCHB.html. 20112019).
17. Williams R. Using the margins command to estimate and interpret adjusted predictions and marginal effects. *Stata Journal* 2012; **12**(2): 308-31.
18. World B. Measuring Living Standards: Household Consumption and Wealth Indices, Quantitative Techniques for Health Equity Analysis-Technical Note #4. 2004. http://siteresources.worldbank.org/INTPAH/Resources/Publications/Quantitative-Techniques/health_eq_tn04.pdf
19. Weber S. A simple command to calculate travel distance and travel time. *The State Journal* 2018; **17**(4): 962-71.
20. Saikia D, Kangkana Das K. Rural Health Infratsutures in the North-East India. Munich: Munich Personal RePEc Archive, 2012.
21. Guilmoto CZ, Dumont A. Trends, Regional Variations, and Socioeconomic Disparities in Cesarean Births in India, 2010-2016. *JAMA Netw Open* 2019; **2**(3): e190526.
22. ICF. The DHS Program STATcompiler 2012. www.statcompiler.com. (accessed 15/1/20 2020).
23. Prusty RK, Choithani C, Gupta SD. Predictors of hysterectomy among married women 15-49 years in India. *Reprod Health* 2018; **15**(1): 3.

For peer review only - http://bmjopen.bmj.com/site/about/guidelines.xhtml

24. Neuman M, Alcock G, Azad K, et al. Prevalence and determinants of caesarean section in private and public health facilities in underserved South Asian communities: cross-sectional analysis of data from Bangladesh, India and Nepal. *BMJ open* 2014; **4**(12): e005982.
25. Boatin AA, Schlotheuber A, Betran AP, et al. Within country inequalities in caesarean section rates: observational study of 72 low and middle income countries. *BMJ* 2018; **360**: k55.
26. Desai G, Anand A, Modi D, et al. Rates, indications, and outcomes of caesarean section deliveries: A comparison of tribal and non-tribal women in Gujarat, India. *PLoS One* 2017; **12**(12): e0189260.
27. Singh P, Hashmi G, Swain PK. High prevalence of cesarean section births in private sector health facilities- analysis of district level household survey-4 (DLHS-4) of India. *BMC Public Health* 2018; **18**(1): 613.
28. Kashanian M, Javadi F, Haghighi MM. Effect of continuous support during labor on duration of labor and rate of cesarean delivery. *Int J Gynaecol Obstet* 2010; **109**(3): 198-200.
29. Lydon-Rochelle M. Cesarean delivery rates in women cared for by certified nurse-midwives in the United States: a review. *Birth* 1995; **22**(4): 211-9.
30. Desai S, Shuka A, Nambiar D, Ved R. Patterns of hysterectomy in India: a national and state-level analysis of the Fourth National Family Health Survey (2015-2016). *BJOG* 2019; **126 Suppl 4**: 72-80.
31. Yadav V, Kumar S, Balasubramaniam S, et al. Facilitators and barriers to participation of private sector health facilities in government-led schemes for maternity services in India: a qualitative study. *BMJ open* 2017; **7**(6): e017092.
32. O'Donnell O. Access to health care in developing countries: breaking down demand side barriers. *Cad Saude Publica* 2007; **23**: 2820 - 34.
33. White MC, Hamer M, Biddell J, et al. Facilitating access to surgical care through a decentralised case-finding strategy: experience in Madagascar. *BMJ Glob Health* 2017; **2**(3): e000427.
34. Ensor T, Bhatt H, Tiwari S. Incentivizing universal safe delivery in Nepal: 10 years of experience. *Health Policy Plan* 2017; **32**(8): 1185-92.
35. De Costa A, Patil R, Kushwah SS, Diwan VK. Financial incentives to influence maternal mortality in a low-income setting: making available 'money to transport' - experiences from Amarpatan, India. *Glob Health Action* 2009; **2**.

For peer review only - http://bmjopen.bmj.com/site/about/guidelines.xhtml

Tables

Table 1: Correlation between surgery, income per capita and supply side variables across all states of India

	Surgical procedures per 1,000 population	C-section rate (% live births)	Hysterectomy per 1,000 women	Sterilisations per 1,000 women
All India	3.1	17%	0.8	2.5
NE India States	2.8	18%	0.5	0.8
**R-squared statistics**				
Registered doctors per 1,000	0.373***	0.612***	0.179**	0.003
Government doctors per 1,000	0.281**	0.234**	0.102*	0.252**
Beds per 1,000	0.288**	0.336**	0.090*	0.094*
GDP per capita	0.103*	0.280**	0.047	0.020

Note: significant at the *** 1% level, ** 5% level, * 10% level.

For peer review only

For peer review only - http://bmjopen.bmj.com/site/about/guidelines.xhtml

Table 2: Association between use of surgical services and individual, household and supply side characteristics (Multivariate logit with cluster adjusted standard errors)

	Caesarean Section			Hysterectomy			Sterilisation		
	Coef.	t-statistic	sig.	Coef.	t-statistic	sig.	Coef.	t-statistic	sig.
**Wealth group (base=poorest)**									
poorer	0.483	3.840	***	0.495	2.480	**	0.013	0.190	
middle	1.093	8.390	***	0.739	3.610	***	0.105	1.380	
richer	1.629	11.660	***	1.162	5.480	***	0.184	2.010	**
richest	2.072	13.340	***	1.330	5.480	***	0.459	3.740	***
	-	-		-	-		-	-	
**Education (base=no education)**	-	-		-	-		-	-	
Completed primary	0.552	3.660	***	0.301	1.900	*	- 0.012	- 0.170	
Completed secondary	0.834	6.370	***	- 0.080	- 0.590		- 0.382	- 5.460	***
Completed Higher	1.230	7.950	***	- 0.253	- 0.980		- 0.931	- 7.100	***
	-	-		-	-		-	-	
**Parity (base=1 delivery)**	-	-		-	-		-	-	
	-	-		-	-		-	-	
Two	- 0.641	- 9.060	***	0.136	0.780		2.432	18.630	***
Three	- 1.291	- 12.190	***	0.162	0.880		2.914	21.090	***
Four or more	- 1.882	- 13.170	***	- 0.145	- 0.780		2.823	19.450	***
	-	-		-	-		-	-	
**Marital status (base=never married)**	-	-		-	-		-	-	
married	- 0.763	- 1.380		1.074	2.400	**	1.078	1.350	
widowed	- 0.875	- 1.360		0.579	1.180		0.424	0.530	
divorced	- 0.694	- 1.050		1.233	2.140	**	0.473	0.560	
living together/separated	- 0.999	- 1.530		0.108	0.170		0.619	0.730	
	-	-		-	-		-	-	
**Religion (base=hindu)**	-	-		-	-		-	-	
muslim	- 0.660	- 5.840	***	- 0.723	- 3.230	***	- 1.649	- 14.340	***
christian	- 0.281	- 2.510	**	- 0.014	- 0.070		- 0.298	- 2.850	***
sikh	3.705	3.090	***	-	-		0.273	0.200	
buddhist/neo-buddhist	- 0.671	- 3.850	***	0.347	0.850		- 0.225	- 1.350	
jain	- 1.851	- 1.320		3.264	2.670	***	1.336	2.190	**
no religion	0.436	0.740		- 0.086	- 0.110		- 1.007	- 2.540	**
other	- 0.111	- 0.860		0.230	1.020		- 1.077	- 9.420	***
	-	-		-	-		-	-	

For peer review only

	Caesarean Section			Hysterectomy			Sterilisation		
	Coef.	t-statistic	sig.	Coef.	t-statistic	sig.	Coef.	t-statistic	sig.
**Caste/Tribe (base=none)**	-	-		-	-		-	-	
Caste	- 0.151	- 1.360		- 0.100	- 0.530		0.165	1.620	
Tribe	- 0.562	- 4.100	***	- 0.259	- 0.970		- 0.148	- 1.150	
	-	-		-	-		-	-	
**Age group (base=15-19)**	-	-		-	-		-	-	
20-25	0.589	3.460	***	- 2.426	- 3.740	***	0.869	1.190	
25-30	0.913	5.260	***	- 1.229	- 4.290	***	1.289	1.800	*
30-35	1.311	7.190	***	0.103	0.510		1.671	2.320	**
35-40	1.589	8.340	***	0.821	5.430	***	1.810	2.500	**
40-45	1.613	6.630	***	0.529	4.360	***	1.715	2.380	**
45-50	1.371	2.550	**	-	-		1.607	2.220	**
								-	
**Type of insurance (base=no insurance)**								-	
state	0.072	0.580		0.017	0.120		0.226	2.550	**
rsby	- 0.254	- 2.170	**	0.092	0.470		0.266	3.550	***
Other insurance	0.252	1.100		0.008	0.020		0.434	2.800	***
	-	-		-	-		-	-	
**Supply-side variables**	-	-		-	-		-	-	
Drs/1,000 population	0.022	1.670	*	- 0.043	- 1.670	*	0.031	3.090	***
Other medical/1,000 population	- 0.021	- 2.930	***	0.011	0.990		- 0.044	- 7.050	***
Time to main surgical facility	- 0.001	- 3.550	***	- 0.001	- 2.340	**	- 0.000	- 0.710	
	-	-		-	-		-	-	
Beds/1,000 population	0.017	3.690	***	0.005	0.720		0.025	6.620	***
	-	-		-	-		-	-	
Constant	- 2.494	- 4.120	***	-	-		- 6.695	- 6.320	***

Note: Significant at the *** 1% level, ** 5% level, * 10% level.

For peer review only

For peer review only - http://bmjopen.bmj.com/site/about/guidelines.xhtml

Table 3: costs of delivery care (US dollars and % of per capita income)

	Public	Private	Total	Public	Private	Total
**Non-surgical delivery**	Cost per delivery			% of per capita income		
Facility	$ 38	$ 140	$ 50	3.3%	12.0%	4.3%
Transport	$ 5	$ 9	$ 6	0.4%	0.8%	0.5%
Total	$ 44	$ 148	$ 56	3.8%	12.8%	4.8%
**Surgical delivery**						
Facility	$ 120	$ 311	$ 199	10.3%	26.8%	17.1%
Transport	$ 8	$ 15	$ 11	0.7%	1.3%	0.9%
Total	$ 128	$ 325	$ 210	11.0%	28.0%	18.1%

Note: state-wise GDP obtained from http://mospi.nic.in/data

Figure legends

Figure 1: Major surgeries per 1,000 population across states of India (2016-17)

Figure 2: Proportion of women that reported undergoing three gynaecological/obstetric procedures

Figure 3: Variation in CS by distance from substantial surgical facility

Figure 4: Variation in hysterectomy (prevalence) by distance from substantial surgical facility

Figure 5: Caesarean section rate as proportion of all deliveries in public and private facilities

For peer review only

For peer review only - http://bmjopen.bmj.com/site/about/guidelines.xhtml

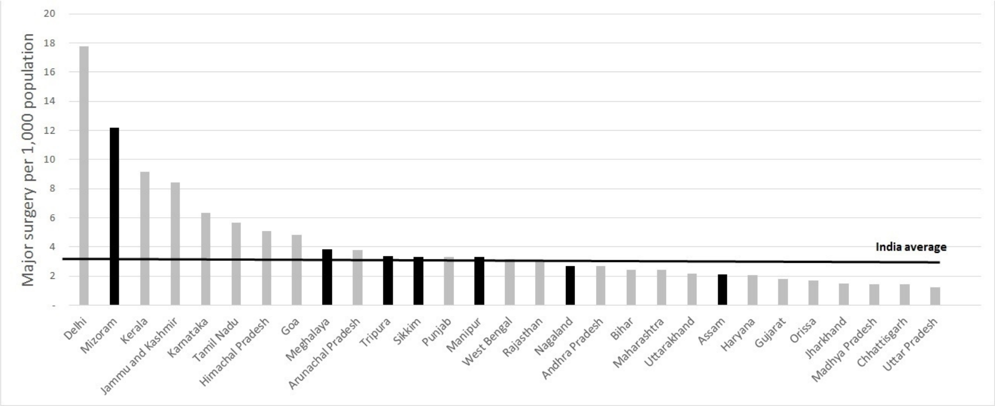

Figure 1: Major surgeries per 1,000 population across states of India (2016-17)

278x115mm (120 x 120 DPI)

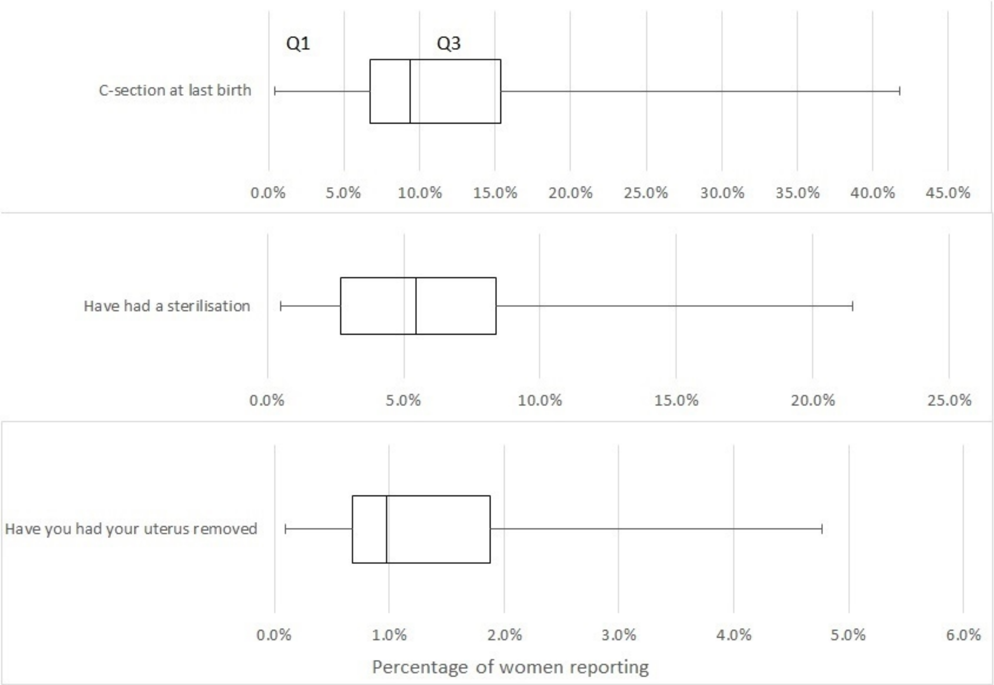

Figure 2: Proportion of women that reported undergoing three gynaecological/obstetric procedures

179x124mm (120 x 120 DPI)

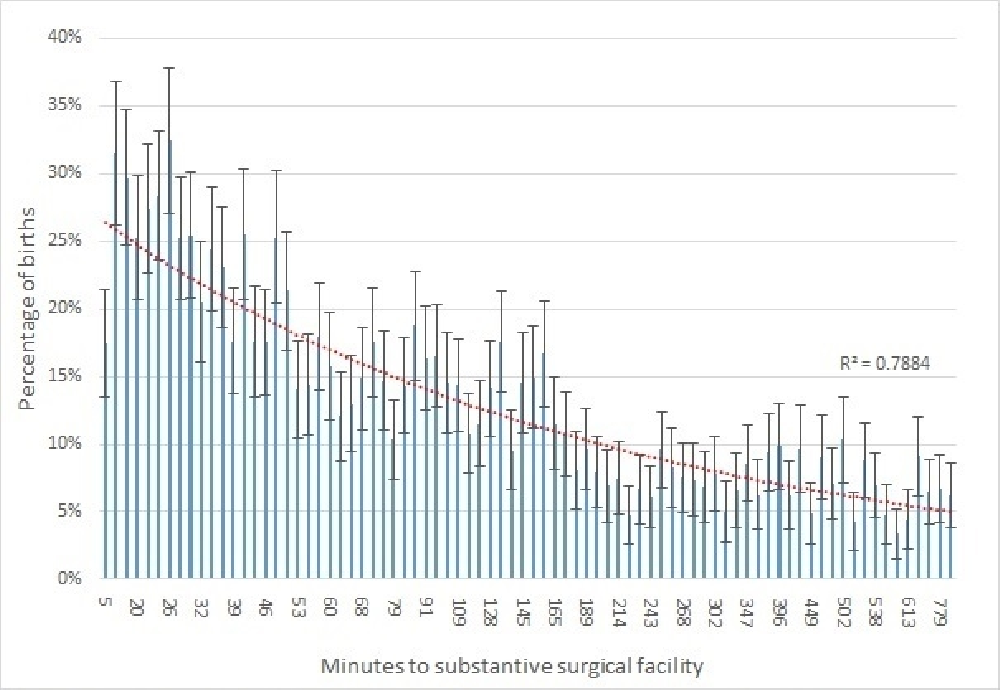

Figure 3: Variation in CS by distance from substantial surgical facility

128x89mm (120 x 120 DPI)

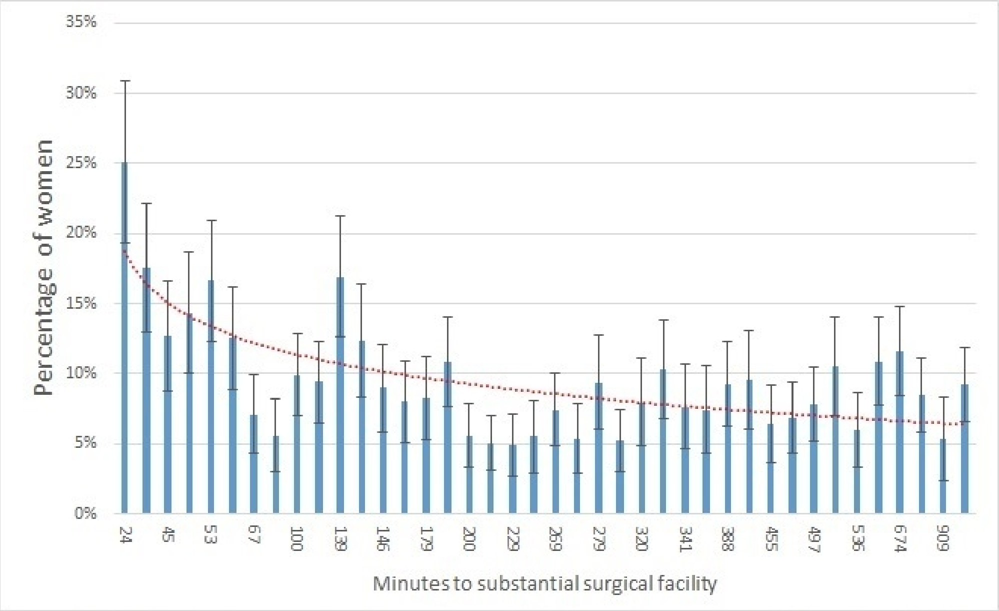

Figure 4: Variation in hysterectomy (prevalence) by distance from substantial surgical facility

139x85mm (120 x 120 DPI)

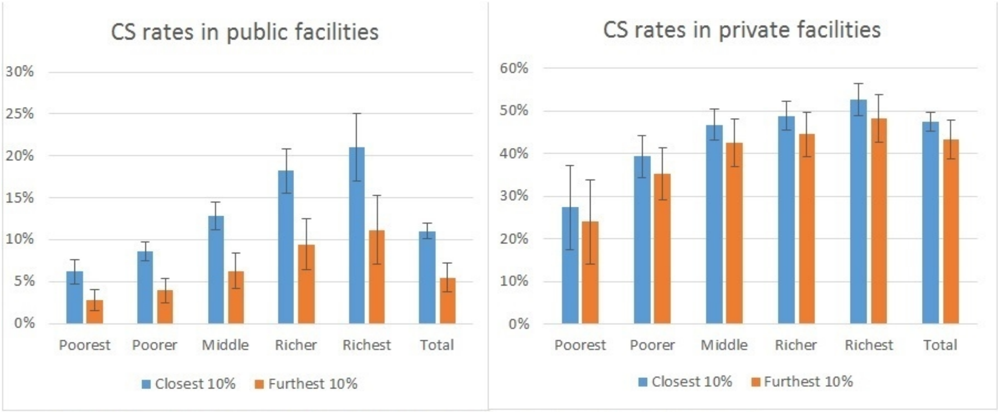

Figure 5: Caesarean section rate as proportion of all deliveries in public and private facilities

190x78mm (120 x 120 DPI)

STROBE Statement—Checklist of items that should be included in reports of ***cross-sectional studies***

	Item No	Recommendation	Page No
**Title and abstract**	1	(*a*) Indicate the study's design with a commonly used term in the title or the abstract	1
		(*b*) Provide in the abstract an informative and balanced summary of what was done and what was found	2
**Introduction**			
Background/rationale	2	Explain the scientific background and rationale for the investigation being reported	4
Objectives	3	State specific objectives, including any prespecified hypotheses	4
**Methods**			
Study design	4	Present key elements of study design early in the paper	4-5
Setting	5	Describe the setting, locations, and relevant dates, including periods of recruitment, exposure, follow-up, and data collection	4
Participants	6	(*a*) Give the eligibility criteria, and the sources and methods of selection of participants	4
Variables	7	Clearly define all outcomes, exposures, predictors, potential confounders, and effect modifiers. Give diagnostic criteria, if applicable	5
Data sources/ measurement	8*	For each variable of interest, give sources of data and details of methods of assessment (measurement). Describe comparability of assessment methods if there is more than one group	*5*
Bias	9	Describe any efforts to address potential sources of bias	NA
Study size	10	Explain how the study size was arrived at	5
Quantitative variables	11	Explain how quantitative variables were handled in the analyses. If applicable, describe which groupings were chosen and why	5
Statistical methods	12	(*a*) Describe all statistical methods, including those used to control for confounding	5
		(*b*) Describe any methods used to examine subgroups and interactions	5
		(*c*) Explain how missing data were addressed	
		(*d*) If applicable, describe analytical methods taking account of sampling strategy	5
		(*e*) Describe any sensitivity analyses	NA
**Results**			
Participants	13*	(a) Report numbers of individuals at each stage of study—eg numbers potentially eligible, examined for eligibility, confirmed eligible, included in the study, completing follow-up, and analysed	4
		(b) Give reasons for non-participation at each stage	NA
		(c) Consider use of a flow diagram	
Descriptive data	14*	(a) Give characteristics of study participants (eg demographic, clinical, social) and information on exposures and potential confounders	Not included due to word length
		(b) Indicate number of participants with missing data for each variable of interest	

For peer review only - http://bmjopen.bmj.com/site/about/guidelines.xhtml

Outcome data	15*	Report numbers of outcome events or summary measures	
Main results	16	(*a*) Give unadjusted estimates and, if applicable, confounder-adjusted estimates and their precision (eg, 95% confidence interval). Make clear which confounders were adjusted for and why they were included	7
		(*b*) Report category boundaries when continuous variables were categorized	7
		(*c*) If relevant, consider translating estimates of relative risk into absolute risk for a meaningful time period	NA
Other analyses	17	Report other analyses done—eg analyses of subgroups and interactions, and sensitivity analyses	8
**Discussion**			
Key results	18	Summarise key results with reference to study objectives	6-8
Limitations	19	Discuss limitations of the study, taking into account sources of potential bias or imprecision. Discuss both direction and magnitude of any potential bias	10
Interpretation	20	Give a cautious overall interpretation of results considering objectives, limitations, multiplicity of analyses, results from similar studies, and other relevant evidence	9-10
Generalisability	21	Discuss the generalisability (external validity) of the study results	10-11
**Other information**			
Funding	22	Give the source of funding and the role of the funders for the present study and, if applicable, for the original study on which the present article is based	11

*Give information separately for exposed and unexposed groups.

Note: An Explanation and Elaboration article discusses each checklist item and gives methodological background and published examples of transparent reporting. The STROBE checklist is best used in conjunction with this article (freely available on the Web sites of PLoS Medicine at http://www.plosmedicine.org/, Annals of Internal Medicine at http://www.annals.org/, and Epidemiology at http://www.epidem.com/). Information on the STROBE Initiative is available at www.strobe-statement.org.

For peer review only

For peer review only - http://bmjopen.bmj.com/site/about/guidelines.xhtml
